# Dissecting the yield architecture and selecting superior seedling-derived half-sib progenies of turmeric (*Curcuma longa* L.) through multi-trait selection indices

**DOI:** 10.3389/fpls.2026.1812796

**Published:** 2026-05-05

**Authors:** Akkurthi Neeraja, S. Mukesh Sankar, Sounderarajan Aarthi, Kotha Madduri Yuvaraj, Duraisamy Prasath

**Affiliations:** 1 Division of Crop Improvement and Biotechnology, Indian Council of Agricultural Research – Indian Institute of Spices Research (ICAR –IISR), Kozhikode, Kerala, India; 2 Dr. Y.S.R. Horticultural University (Dr. YSRHU) College of Horticulture, Anantharajupeta, Andhra Pradesh, India; 3 ICAR –All India Coordinated Research Project on Spices (AICRPS), ICAR-IISR, Kozhikode, Kerala, India

**Keywords:** *Curcuma longa* (Linn.), Genetic parameters, Half-sib progenies, Selection models, Source–sink relationships, Structural equation modeling (SEM), Yield architecture

## Abstract

Turmeric (*Curcuma longa* L.), propagated vegetatively, harbors limited genetic variability for breeding, prompting exploration of rare seed-derived half-sib progenies to generate novel recombinants. The present study investigated 263 first-generation seedling-derived half-sib progenies from five maternal accessions alongside three checks in an augmented randomized complete block design over two years (2024–2025) at Kozhikode, India. Significant genotypic variation was observed across 17 vegetative, rhizome, yield, and percentage dry recovery, with high broad-sense heritability (60–97%) for most, including total yield per plant (80.73%), primary rhizome weight (64.3%), and dry recovery (95.73%). Genotypic coefficients of variation exceeded 20% for key yield components, indicating strong selection potential. Pearson correlations highlighted strong positive associations of total yield with primary rhizome weight (r = 0.81, p< 0.01), mother rhizome weight (r = 0.60), and primary rhizome number (r = 0.57), alongside a yield–dry recovery trade-off (r = -0.20). Stepwise regression identified primary rhizome weight as the strongest predictor (adjusted R² = 0.791). Structural equation modeling confirmed vegetative growth (measured by leaf width, leaf number, petiole length) strongly influences rhizome sink strength (β = 0.860, p = 0.027), mediating indirect effects on total yield per plant. Multi-trait indices—multi-trait genotype-ideotype distance (MGIDI), multi-trait stability (MTSI), Smith-Hazel (SH), and factor analysis-ideotype (FAI)—identified 53 superior progenies (20% intensity), with 14 common selections across methods, prioritizing balanced yield, sink capacity, and stability. These findings validate seedling progenies as a practicable resource for turmeric improvement, offering observable vegetative proxies for subterranean yield traits and ideotype-based frameworks to accelerate recombination breeding.

## 1 Introduction

Turmeric (*Curcuma longa* L.), a perennial herbaceous plant from the Zingiberaceae family, has been cherished for centuries for its diverse applications in traditional medicine, culinary practices, and cultural rituals. Contemporary pharmacological studies have reinforced many of these traditional claims, elevating turmeric to the status of a globally important natural therapeutic agent (Prasath et al., 2024). India remains the world’s largest producer and exporter of turmeric, contributing nearly 80% of the global supply. In India, turmeric is cultivated over an area of 0.29 million hectares with a production of 11.16 lakh tons during 2024 –2025. Notably, Maharashtra, Telangana, Karnataka, Tamil Nadu, Madhya Pradesh, Andhra Pradesh, and Odisha, which together, holds major acreage for turmeric cultivation, contributing significantly to the national and global supply (Spices Board, 2026).

Botanically, turmeric is adapted for insect-mediated cross-pollination, but exists predominantly as a triploid (2n = 3x = 63) and, less frequently, as a tetraploid (2n = 4x = 84), reproducing almost exclusively through vegetative propagation *via* clonal rhizomes (Aswathi and Prasath, 2024). Flowering in turmeric typically occurs only after the plant and its rhizome reach sufficient maturity and resource status. Warm and humid tropical conditions favors flower development, and field reports indicate that flowering commonly appears within a window of ~109–155 days after planting, depending on cultivar and environmental conditions. Flowering is more likely on vigorous plants with large mother rhizomes and abundant tillers. Controlled studies show that night-interruption/long-day regimes induce flowering in related species (Flores et al., 2021; Deng et al., 2023; Stirling et al., 2002) and can alter flowering and assimilate partitioning to storage organs in turmeric, although the response is varies among genotypes. Turmeric flowers are normally sterile, and viable seed set is rare in most cultivars (Nayar, 2014), primarily due to ploidy-associated meiotic abnormalities, pollen sterility and poor pollen–stigma interaction and female gametophytic dysfunction, asynchronous and sporadic flowering, self-incompatibility, and early seed abortion. As a result, practical artificial hybridization programs have been largely ineffective and turmeric breeding has historically relied on induced mutation and clonal selection for genetic improvement (Sankar et al., 2025). Although accumulated somatic mutations in the clonal populations generate considerable morphological diversity, the absence of sexual recombination limits realized genetic variability available for selection is limited. Molecular marker surveys have nonetheless revealed substantial polymorphism in turmeric germplasm, indicating hidden genetic diversity (e.g. 82% polymorphism among accessions), but that diversity has yet to be effectively harnessed together into improved cultivars (Verma et al., 2015).

Turmeric cultivation is constrained by several interconnected challenges that directly influence productivity and varietal adoption. These include low genetic variability resulting from prolonged clonal propagation, heightened susceptibility to rhizome rot and leaf blotch diseases, and significant yield instability across environments due to strong genotype × environment interactions (Ravindran et al., 2007).

**Figure 1:**
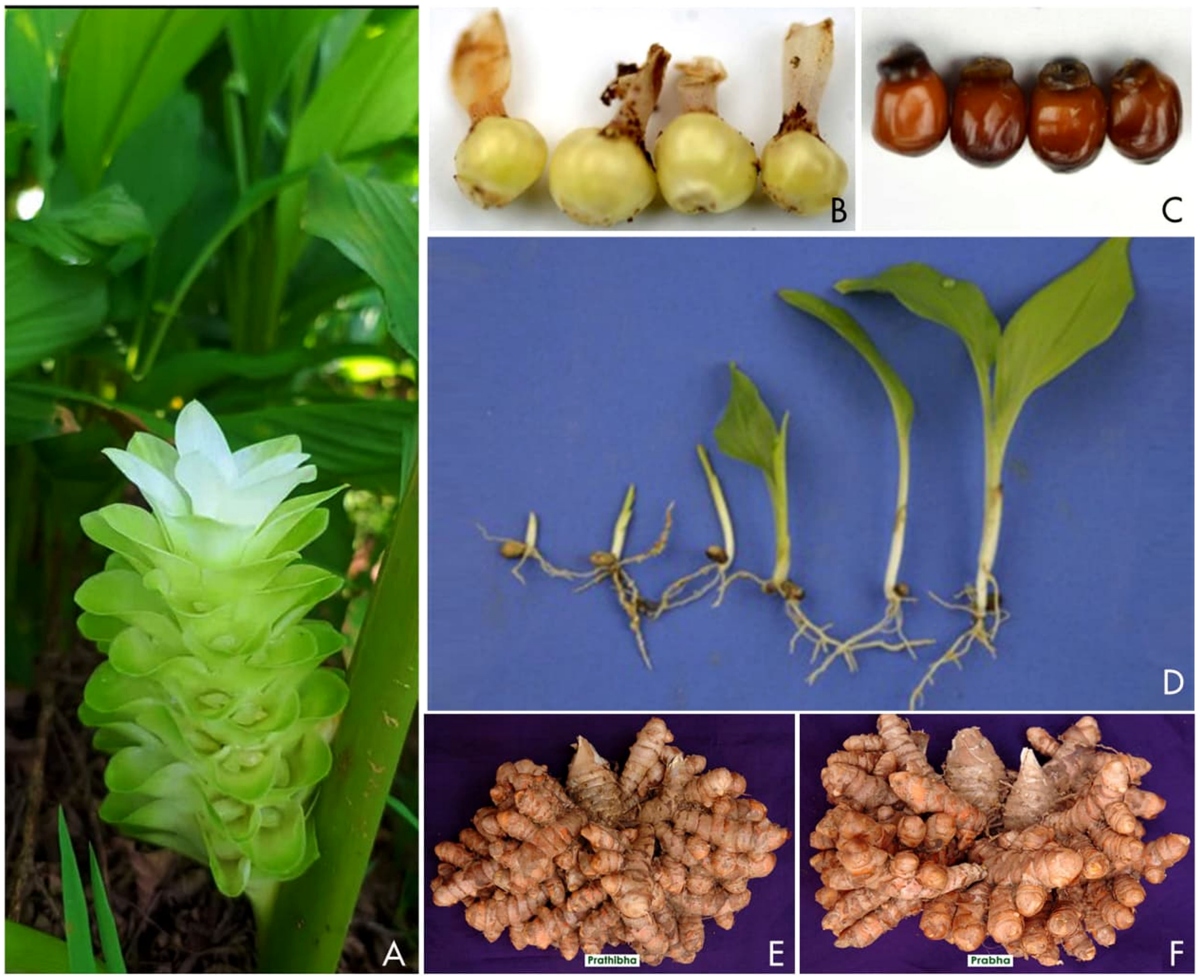
Scenario of seed-based life cycle of turmeric **(A)** Inflorescence **(B)** Capsules **(C)** Seeds **(D)** Seedling development, **(E, F)** Varieties derived from seedling progenies.

One underexplored strategy to create new variability and subsequently recombine it in turmeric is to exploit the rare instances of seed production. While most cultivated turmeric cultivars are effectively sterile, viable seed sets can occur under certain conditions. Sasikumar (2005) and others have noted that “viable seed sets obtained in certain cases enable recombination breeding through hybridization and open-pollinated progeny”. In practice, inter-varietal crosses and open-pollinated seedlings have been produced in research settings. The resulting capsules (fruit) with a few recalcitrant seeds, some of which germinate slowly after several months. Similarly, open-pollinated progenies from selected plants have yielded germinated seedlings, although germination is often erratic and protracted. This approach still shows promise. For instance, ‘IISR Prabha’ and ‘IISR Prathibha’ (**Figure 1**) are among the first turmeric varieties developed at ICAR-IISR through open-pollinated progeny selections (Sasikumar et al., 1996). These varieties possess high-curcumin content and higher yield, are preferred by farmers. Although limited, these studies demonstrate that turmeric can set seeds and generate first-generation recombinants suggesting the potential for breeding programs based on seed-derived variation. To date, such approaches have not been pursued systematically.

Within this context, half-sib progenies represent a particularly valuable genetic resource for perennial and clonally propagated crops. A half-sib family comprises individuals that share a common maternal parent but differ in paternal parentage due to open pollination (Tahir et al., 2024). Although true seed formation is rare in turmeric, half-sib progenies can be generated through controlled pollination or natural outcrossing, providing a structured population for genetic evaluation. Assessment of half-sib progenies enables the estimation of additive genetic variance, heritability, and selection potential, parameters that are critical for crop improvement (Meenakshi et al., 2021; Aswathi et al., 2023; Raghuveer et al., 2023). This strategy allows breeders to exploit within-family variability while retaining favorable maternal attributes, thereby providing a viable pathway for the developing improved genotypes.

However, the estimation of variance components alone is insufficient for effective selection, as yield is a complex trait governed by interactions among multiple component traits. While correlation analysis identifies traits that vary together, it does not elucidate causal relationships. Trait association studies, including multiple regression and path coefficient analyses, further assist in identifying key yield components and managing trade-offs among correlated traits (Singh and Chaudhary, 1981). Recent advances in genotype-to-phenotype modeling and quantitative prediction frameworks have further enhance breeding efficiency by enabling the simulation of complex trait interactions across environments (van Eeuwijk et al., 2019). Structural equation modeling (SEM) provides a robust framework to disentangle direct and indirect effects of component traits on total rhizome yield. SEM explicitly represents hypothesized causal pathways and quantifies their relative contributions. In plant breeding, SEM has been applied to characterize cascades of trait effects, such as linking quantitative trait loci to yield through intermediate components in cereals (Lamb et al., 2011; Li et al., 2019; Kapoor et al., 2022) and in spices (Chinnathambi et al., 2024). By constructing SEMs that incorporate latent constructs for vegetative growth (*e.g.*, plant height, number of leaves) and sink strength (e.g., rhizome number and weight), the relative contributions of these trait complexes to final yield can be quantified. Compared with conventional path analysis, SEM accommodates multiple interrelated pathways and is particularly suited to resolving complex trait networks in non-model, vegetatively propagated crops such as turmeric. Integrating classical quantitative genetics with predictive modeling therefore offers a powerful approach to accelerate genetic improvement despite biological constraints.

Finally, the identification of superior progenies in turmeric requires integrative selection strategies that can simultaneously account for multiple, often correlated traits. Accordingly, the present study employed multivariate selection indices to rank genotypes holistically rather than on the basis of individual traits. Conventional indices such as the Smith–Hazel (SH) index, which rely on economic weights and phenotypic–genotypic covariance matrices, are known to be sensitive to multicollinearity and to inaccuracies in covariance estimation, particularly when many traits are measured. Recent advances in selection index methodology have addressed these limitations through ideotype-based and factor-analytic approaches. Among these, the Multi-trait Genotype–Ideotype Distance Index (MGIDI) defines a hypothetical ideotype with optimal trait values and ranks genotypes based on their multivariate distance from this ideotype, thereby enabling balanced selection across traits (Olivoto and Nardino, 2021). By employing factor analysis, MGIDI transforms correlated traits into orthogonal latent factors representing distinct biological dimensions such as yield, morphology, and quality, facilitating the explicit evaluation of trade-offs among trait groups (Rocha et al., 2018). Additionally, the Factor Analysis and Ideotype-Design (FAI) index and the Multi-Trait Stability Index (MTSI) were employed to complement MGIDI, with MTSI integrating both mean performance and stability across environments. The SH index was retained for comparison, and a coincidence index was used to quantify consistency among selection methods. Considering the recombination-derived variability observed in seedling-derived half-sib progenies and the complex, multivariate nature of economically important traits in turmeric, the combined application of MGIDI and related factor-based indices provides a robust and unbiased framework for genotype evaluation. This integrative approach is expected to improve selection accuracy and accelerate the identification of high-performing turmeric genotypes suitable for diverse agro-climatic conditions and industrial end uses.

Against this backdrop, the present study adopts a novel and integrative approach to improve turmeric rhizome yield by tapping the genetic potential of seedling progenies and employing rigorous statistical analyses to understand trait interplay. Using a large population of half-sib progenies derived from multiple maternal parents, the study aimed to (i) quantify genetic variability and key genetic parameters generated through sexual recombination for important morphological and rhizome traits, (ii) elucidate the hierarchical relationships among yield-contributing traits in seedling progenies through structural equation modelling, and (iii) identify superior seedling progenies for rhizome yield using multiple complementary multi-trait selection indices. By combining classical quantitative genetics with contemporary statistical and modelling approaches, this study seeks to provide a robust framework for recombination-based turmeric breeding and to demonstrate the feasibility of integrating seedling progeny evaluation into mainstream improvement programs.

## 2 Materials and methods

A total of 257 first-generation seedling-derived half-sib progenies (F_1_-equivalent from open pollination) followed by four successive generation of clonal multiplication along with their five maternal parents (*Acc.* 19–177, *Acc.* 138–53, *Acc.* 1234–16, *Acc.* 274–8, and *Acc.* 320–3) and three check varieties (IISR Pragati, IISR Alleppey Supreme, and IISR Suguna) were evaluated using an augmented randomized complete block design (ARCBD) comprising 14 blocks, with the checks replicated in each block. The detailed pedigree of genotypes is provided in supplementary file 1. The field experiment was conducted at the ICAR-Indian Institute of Spices Research, Experimental Farm, Kozhikode, Kerala (11°36’34’’ N; 75°49’12’’ E; 60 m MSL) during the year 2024–2025 following the experimental design layout generated using “FielDHub”R based Shiny app (Murillo et al., 2021). Planting was carried out in *kharif* season using healthy, disease-free rhizome bits in uniformly prepared beds measuring 3 × 1 m. Each bed accommodated three accessions, with 12 plants per genotype, maintained at a spacing of 30 × 25 cm. Standard agronomic practices, including timely irrigation, balanced fertilization, and appropriate pest and disease management measures, were followed to ensure optimum crop growth and establishment (Prasath et al., 2022).

Observations on morphological and yield traits were recorded from five representative plants per plot following DUS guidelines (PPV&FRA, 2009) for turmeric. Plant height (PH) was measured from the base to the tip of the plant. Total tillers per plant (NT) and leaf number (NL) were recorded by counting all tillers and leaves of each sampled plant. Leaf lamina length (LL) and width (LW) were measured from the leaf base to the tip and at the widest portion of the lamina, respectively, and leaf area (LA) was calculated from lamina length and width using an appropriate correction factor. Collar girth (CG) was measured by placing a thread around the pseudostem at the collar region, while petiole length (PL) was measured from the top of the leaf sheath to the base of the blade. For yield-related traits, the weight of mother rhizomes (WMR), the weight of primary rhizomes (WPR) and the number of mother (NMR) and primary (NPR) rhizomes were recorded for each clump. The length of primary rhizomes (LPR) was measured from the point of attachment to the mother rhizome to the tip and the girth of the primary rhizome (GPR) was measured at the midpoint using a vernier caliper. Internodal length (IL) on primary rhizomes was determined as the distance between two successive nodes. Total yield (TY) was computed by weighing the total rhizome mass per plant. Dry recovery (DR) was determined by collecting all rhizomes from each plant, boiling them and drying them under sunlight to constant weight, and subsequently recording the dry weight.

All statistical analyses were performed in R version 4.3.2. The analysis of the Augmented Randomized Complete Block Design (ARBD) was conducted using the ‘augmentedRCBD’ package (Aravind et al., 2021) to obtain the ANOVA results and adjusted means for each genotype.

The linear model for the ARBD is expressed as:


$$Y_{ij(k)}=\mu +\beta_{i}+C_{j}+\tau k_{\mathrm{(i)}}+\epsilon_{ij}$$

Where,

Y_ij(k)_ is the observed value of the j^th^ check or k^th^ entry in the i^th^ block, μ is the overall mean, β_i_ is the effect of i^th^ block, C_j_ is the effect of j^th^ check treatment, τ_k(i)_ is the effect of the k^th^ entry in the i^th^ block and ϵ_ij_ is the random error associated with the observation.

Adjusted means were computed following the procedure described by Federer (1956, 1961), and the resulting block-adjusted values for each environment were used in all subsequent analyses. The adjusted mean of each block was calculated with the formula (Federer, 1961):


$$V_{i}=\mu_{i}-b_{j}$$

Where,

V_i_ is the adjusted mean of *i^th^* variety.

*u_i_* is the unadjusted mean of *i^th^* variety.

*b_j_* is *j^th^* block effect.

Variance components, including genotypic variance (σ_g_^2^), phenotypic variance (σ_p_^2^) and error variance (σ_e_^2^), were estimated from the expected mean squares of ANOVA described by Federer and Searle (1976) as follows:

σ_p_^2^= Mean sum of squares of test treatments;

σ_e_^2^ = Mean sum of squares of residuals.


$$\sigma_{g}^{2}=\;\sigma_{p}^{2}-\;\sigma_{e}^{2}$$

Prior to pooled analysis, Bartlett’s Chi-square test was performed to assess the homogeneity of variances across environments. The results (p > 0.05) confirmed homogeneity, validating the pooled analysis. The combined analysis across years was conducted using a linear mixed modelling framework, which allows flexible treatment of genotypic and interaction effects and provides efficient estimation of variance components under unbalanced data structures. For each trait, the following model was fitted:


Yij=μ+Gi+Ej+(G×E)ij+ϵij

Where, Y_ij_ is the adjusted mean of the i^th^ genotype in the j^th^ year, μ is the overall mean, Gi is the effect of the i^th^ genotype, E_j_ is the effect of the j^th^ environment (year), (G×E)ij is the genotype × environment interaction effect, and ϵij is the residual error term associated with the adjusted means.

In the mixed model, genotypes and genotype × environment interactions were treated as random effects, while years were treated as fixed effects, reflecting the specific nature of the two test seasons. Variance components for genotypes, environments, and GEI were estimated using restricted maximum likelihood (REML). The significance of fixed effects and the relative contribution of random effects were assessed through analysis of variance based on the fitted mixed model using the ‘*lme4’* and ‘*lmerTest’* packages in R. Combined genotype performance across years and year-specific genotype responses were obtained from the model as estimated marginal means, which were subsequently used for interpretation of GEI and for downstream selection analyses.

Phenotypic and genotypic coefficients of variation (PCV and GCV) were estimated according to Burton (1951, 1952) using expressions:


$$Genotypic\;coefficient\;of\;variation\;\left(GCV\right),GCV=\frac{\sigma_{g}^{2}}{\sqrt{\bar{x}}}\times 100$$

$$Phenotypic\;coefficient\;of\;variation\;\left(PCV\right),PCV=\frac{\sigma_{p}^{2}}{\sqrt{\bar{x}}}\times 100$$

Where, x̄ is the mean. The magnitude of GCV and PCV was classified according to Madhavamenon and Sivasubramanian (1978), wherein values < 10% were considered low, 10–20% as moderate, and >20% as high.

The broad-sense heritability (H^2^) was calculated according to the method of Lush (1940) as follows:


$$H^{2}=\;\sigma_{g}^{2}/\sigma_{p}^{2}$$

Heritability estimates were categorized following Robinson (1966) as low (<30%), moderate (30–60%), or high (>60%). Genetic advance (GA) was estimated and categorized according to Johnson et al. (1955) as follows:


$$Genetic\;advance\;\left(GA\right),\;GA\;=\;k\;\times \;\sigma_{g}\times \;H^{2}$$

Where the constant k is the standardized selection differential (selection intensity). The value of k at a 5% proportion selected is 2.063.

Trait associations were estimated using standardized values of traits derived from the adjusted means obtained through ARCBD ANOVA. Pearson’s correlation coefficients were computed in R using the ‘car’ (Fox et al., 2007) and ‘corrplot’ package (v0.92) (Wei and Simko, 2010). To identify the most influential agronomic traits with respect to total rhizome mass per plant as a dependent variable, while remaining traits were treated as potential predictors, a stepwise regression model was fitted. Predictor variables were entered sequentially based on improvements in adjusted coefficient of determination (adjusted R²) and reductions in residual standard error, with Akaike Information Criterion (AIC) used as the primary criterion for model selection and parsimony. Multicollinearity among predictors was assessed using variance inflation factors. Model robustness and predictive performance were evaluated using repeated 10-fold cross-validation, with root mean square error (RSME), mean absolute error (MAE), and cross-validated R² used as evaluation metrics.

Structural equation modelling (SEM) was employed to elucidate the directional and hierarchical relationships among key traits influencing yield. Based on regression results and biological considerations, a hypothesized SEM was constructed comprising latent constructs representing vegetative growth and rhizome sink strength. Structural paths were specified from vegetative growth to rhizome sink and from both latent constructs to total yield. SEM analyses were performed using the ‘*lavaan’* package in R. Model adequacy was evaluated using multiple goodness-of-fit indices, including the chi-square (χ²) test, goodness-of-fit index (GFI), and standardized root mean square residual (SRMR), following commonly accepted threshold criteria. These indices were used to assess the consistency between the observed and model-implied covariance structures.

Multi-trait selection was performed using four complementary indices, namely the Multi-trait Genotype–Ideotype Distance Index (MGIDI), Multi-trait Stability Index (MTSI), Smith–Hazel (SH) index, and Factor Analysis and Ideotype-based selection (FAI) following (Olivoto and Nardino, 2021) and Rocha et al. (2018) using the influential trait identified. A selection intensity of 20% (SI = 20) was used to identify the top-performing genotypes based on indices were conducted using the *metan* package in R, while the Smith–Hazel index was computed using phenotypic and genotypic variance–covariance matrices. Additionally, the ‘coincidence index’ function was used to calculate number of common genotypes selected in different multi-trait indices.

## 3 Results

### 3.1 Mean performance and variation for phenotypic traits

The pooled ANOVA revealed highly significant (p< 0.05) differences among genotypes for all growth and yield traits except internodal length, which was non-significant (**Table 1**). Genotypic variation was evident across vegetative descriptors including plant height, tiller number, leaf number, leaf length, leaf area, collar girth and petiole length, as well as major rhizome traits such as mother rhizome number and weight, primary rhizome number, length, girth and weight, and total yield. The test and test vs. check effects were significant for most traits, confirming the presence of meaningful phenotypic divergence among half-sib progenies and the check varieties. Block effects were generally non-significant for many traits, highlighting adequate experimental precision in the augmented design. The significant variation observed across vegetative and rhizome traits confirms the suitability of the experimental material for multi-trait selection.

**Table 1:** Analysis of variance (ANOVA) for vegetative, yield and yield related traits of turmeric half-sib seedling progenies in the augmented design during *Kharif* 2024 and 2025.

Source of variation	df	PH (cm)	NT	NL	LL (cm)	LW (cm)	LA (cm^2^)	PL (cm)	CG (cm)	NMR	WMR (kg)	NPR	LPR (cm)	IL (cm)	GPR (cm)	WPR (kg)	TY (kg)	DR (%)
Genotypes (Ignore block)	264	151.9 **	0.4 **	0.6 **	35.2 **	2.8 **	11,133 **	16.9 **	1.4 **	1.2 **	0.002 **	16.26 **	1.9 **	0.02 NS	13.1 **	0.018 **	0.05 **	15.10 **
Genotypes: Check	2	606.7 **	0.1 NS	0.8 *	134.7 **	5.4 *	112,479 **	86.3 **	0.9 NS	3.9 **	0.049 **	90.75 **	0.81 **	0.38 **	242.9 **	0.003 NS	1.38 **	98.29 **
Genotypes: Seedling Progenies with mother parents	262	131.1 **	0.4 **	0.6 **	34.6 **	2.6 NS	10,286 **	10.7 **	1.5 **	1.2 **	0.001 **	15.38 **	1.89 **	0.02 NS	5.62 **	0.004 **	0.03 **	11.23 **
Genotypes: Seedling progenies with mother parents vs Check	1	4665.4 **	0.6 NS	6.2 **	3.4 NS	40.7 **	29,501 **	1522 **	1.27 NS	2.0 **	0.169 NS	95.10 **	32.44 **	0.02 NS	1519.9 **	3.65 **	5.30 **	859.75 **
Block (Elim. genotypes)	13	5.70 NS	0.62 **	0.68 **	23.4 **	3.2 NS	837.4 NS	7.1 **	0.9 NS	0.16 NS	0.001 NS	0.69 NS	0.16 **	0.03 NS	7.16 **	0.001 NS	0.01 NS	0.59 NS
Residuals	30	14.41	0.20	0.17	6.41	1.66	1212	3.26	0.47	0.12	0.000	0.91	0.05	0.03	1.34	0.001	0.01	0.48

Where, NS denote non-significant and * & ** denotes significant at p<0.05 and p<0.01 respectively; PH, Plant height (cm); NT, Number of tillers plant^-1^; NL, Number of leaves shoot^-1^; LL, Leaf length (cm); LW, Leaf width (cm); LA, Leaf area (cm²); PL, Petiole length (cm); CG, Collar girth (cm); NMR, Number of mother rhizomes plant^-1^; WMR, Weight of mother rhizomes clump^-1^ (kg); NPR, Number of primary rhizomes clump^-1^; LPR, Length of primary rhizome (cm); IL, Internodal length (cm); GPR, Girth of primary rhizome (cm); WPR, Weight of primary rhizome clump^-1^ (kg); TY, Total yield (kg/plant); DR (%), Dry recovery (%).

**Figure 2:**
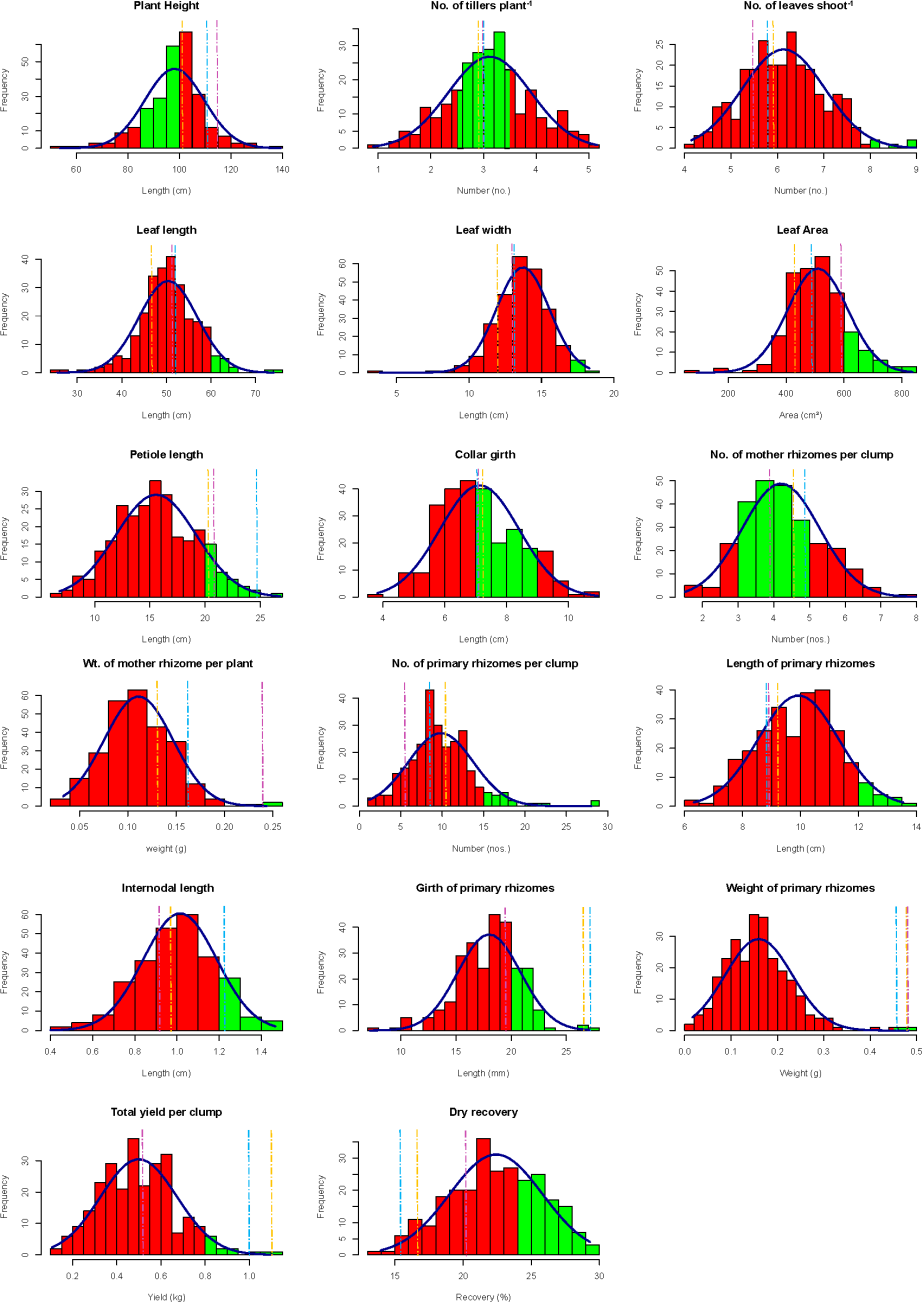
Histograms depicting the distribution of agro-morphological traits among 265 turmeric seedling-derived genotypes evaluated over two consecutive years (2024–25). Red bars represent the observed frequency distribution with an overlaid fitted normal curve (blue). Green-shaded regions indicate the predefined ideotypic range for each trait. Vertical dashed lines denote the performance of check varieties: Yellow (IISR Pragati), Blue (Suguna), and Violet (IISR Alleppey Supreme).

The pooled analysis of two consecutive years (2024–25) revealed substantial phenotypic variability among turmeric seedling-derived genotypes for all agro-morphological, rhizome, yield, and quality trait-dry recovery percentage evaluated across 265 turmeric genotypes (**Table 2**). The distribution of these traits, along with the proportion of genotypes falling within the predefined ideotypic range, is illustrated using histograms (**Figure 2**), where idiotypic classes are highlighted in green shades. Plant height ranged from 55.0 to 128.75 cm, with a mean of 98.15 cm, indicating considerable variability and scope for selection towards optimal plant stature. A total of 125 genotypes fell within the predefined ideotypic range, reflecting the predominance of moderate plant architecture in the population. Tiller number per plant exhibited pronounced variability (range: 1.75–5.50; mean: 3.12), with approximately 207 genotypes approaching the ideotypic range of 4–6 tillers. Leaf-related traits also showed wide variation, including number of leaves per shoot (4.25–8.75), leaf length (25.75–71.75 cm), leaf width (4.75–18.50 cm), and leaf area (145.57–834.79 cm²), indicating diversity in plant architecture and photosynthetic capacity. Petiole length ranged from 6.75 cm (genotype 19×90) to 24.83 cm (cv. Suguna), with a mean of 15.56 cm, reflecting variation in canopy structure and leaf display. Rhizome traits exhibited even greater variability. The number of mother rhizomes ranged from 1.25 to 8.00, with ideotype-oriented selection favoring fewer but bolder mother rhizomes recorded in 213 progenies and mother rhizome weight ranges from 0.024 to 0.279 kg plant^-1^. The number of primary rhizomes per clump ranged from 1.25 to 22.75, while primary rhizome weight varied substantially (0.019 – 0.549 kg plant^-1^.). Total fresh yield spanned a wide interval, nearly six-fold from 0.19 kg (genotype Acc.138-24) to 1.18 kg (genotype Acc.19-121), with a mean of 0.50 kg plant^-1^. Notably, 38 genotypes exceeded the population mean while also approaching ideotypic thresholds. Dry recovery, a key quality determinant, ranged from 14.20% (genotype Acc.19-90) to 28.80% (genotype Acc.19-70), with a mean of 22.41%, indicating substantial variability in rhizome dry matter accumulation. A total of 88 genotypes surpassed the critical 24% dry recovery threshold.

**Table 2:** Overview of agro-morphological traits recorded during the trials.

Parameters	Mean	Range				CD	SE (m)	CV (%)	No. of genotypes fall under Ideal type*
		Min. value	Genotypes	Max. value	Genotypes				
Plant height (cm)	98.15	55	19*159	128.75	19*121	12.67	6.20	3.81	125
No. of tillers plant^-1^	3.12	1.75	19*204, 19*205, 19*228, 138*32, 138*66, 138*67	5.5	19*125	1.5	0.74	14.56	207
No. of leaves shoot^-1^	6.13	4.25	138*38	8.75	19*5	1.38	0.68	6.8	2
Leaf length (cm)	50.39	25.75	19*159	71.75	19*5	8.44	4.13	5.02	11
Leaf width (cm)	13.72	4.75	274*1	18.5	19*70	4.3	2.1	9.46	1
Leaf area (cm^2^)	509.41	145.57	19*159	834.79	19*56	116.14	56.87	6.83	48
Collar girth (cm)	7.12	4	19*90	10.6	138	2.3	1.12	9.59	135
Petiole length (cm)	15.56	6.75	19*90	24.83	Suguna	6.02	2.95	11.01	54
No. of mother rhizomes plant^-1^	4.2	1.25	138*82,19*113, 138*24	8	1234*10	1.16	0.57	8.23	213
Weight of mother rhizomes clump^-1^ (kg)	0.11	0.024	138*82	0.279	A.Supreme	0.07	0.03	17.66	16
No. of primary rhizomes clump^-1^	9.84	1.25	138*72, 1234*2	22.75	19*82	3.12	1.57	9.99	20
Length of primary rhizome (cm)	9.93	6.68	19*158	13.74	19*85	0.76	0.37	2.31	15
Internodal length (cm)	1.02	0.5	19*28	1.5	138*70	0.54	0.27	16.05	30
Girth of primary rhizome (cm)	18.01	8.08	19*93	27.13	Suguna	3.9	1.9	6.14	76
Weight of primary rhizome clump^-1^ (kg)	0.16	0.019	1234*2	0.549	A.Supreme	0.13	0.06	19.35	30
Total yield (kg/plant)	0.5	0.19	138*24	1.18	19*121	0.24	0.12	13.29	38
Dry recovery (%)	22.41	14.2	19*90	28.8	19*70	2.31	1.13	3.17	88

CD, Critical Difference at 5% level of significance; SE(m), Standard Error of the Mean; CV (%), Coefficient of Variation.

*The ideal values for agromorphological traits were derived from previous studies reported by Nambiar et al. (1982); Ravindran et al. (2007), and Kandiannan et al. (2015).

Frequency distribution histograms (**Figure 2**) overlaid with predefined ideotypic ranges as suggested by Nambiar et al. (1982); Ravindran et al. (2007); and Kandiannan et al. (2015) indicated, most traits exhibited approximately normal to slightly right-skewed distributions, particularly for yield-related traits such as total yield, primary rhizome weight, and dry recovery. Genotypes falling within ideotypic windows consistently clustered toward the higher performance classes and were clearly distinguishable from check varieties, supporting the ideotype-guided selection framework for turmeric improvement. Collectively, the pooled results across two years demonstrate that seedling-derived turmeric populations harbor substantial exploitable variability across vegetative, rhizome, yield, and dry recovery percentage, thereby validating the use of ideotype-driven, multi-trait selection strategies for identifying superior recombinants suitable for further advancement.

### 3.2 Estimation of heritability and genetic parameters

The phenotypic variance observed among the turmeric half-sib seedling progenies was partitioned into genotypic variance (σ²g), phenotypic variance (σ²p), along with the corresponding genotypic coefficient of variation (GCV) and phenotypic coefficient of variation (PCV) (**Table 3**). Across vegetative and rhizome yield–related traits, considerable variability was evident, indicating substantial scope for genetic improvement within the progeny population.

**Table 3:** Assessment of heritability and other genetic parameters for vegetative and yield related traits of turmeric half-sib seedling progenies.

Trait	Mean	Phenotypic variance	Genotypic variance	GCV (%)	PCV (%)	H^2^
Plant height (cm)	98.15	131.11	116.70	11.00	11.66	89.01
No. of tillers plant^-1^	3.12	0.44	0.24	15.58	21.28	53.64
No. of leaves shoot^-1^	6.13	0.59	0.41	10.45	12.44	70.60
Leaf length (cm)	50.39	34.58	28.17	10.52	11.66	81.47
Leaf width (cm)	13.72	2.60	0.94	7.05	11.73	36.19
Leaf area (cm^2^)	509.41	10286.19	9073.50	18.65	19.86	88.21
Collar girth (cm)	7.12	1.47	1.00	14.00	16.98	67.96
Petiole length (cm)	15.56	10.69	7.43	17.51	20.99	69.54
No. of mother rhizomes plant^-1^	4.2	1.19	1.07	24.63	26.00	89.72
Weight of mother rhizomes clump^-1^ (kg)	0.11	0.001	0.0008	22.29	29.30	57.88
No. of primary rhizomes clump^-1^	9.84	15.38	14.46	38.82	40.03	94.02
Length of primary rhizome (cm)	9.93	1.89	1.84	13.64	13.83	97.27
Internodal length (cm)	1.02	0.02	NA	NA	14.46	NA
Girth of primary rhizome (cm)	18.01	5.62	4.28	11.46	13.14	76.14
Weight of primary rhizome clump^-1^ (kg)	0.16	0.004	0.003	32.44	40.46	64.3
Total yield (kg/plant)	0.5	0.03	0.02	29.82	33.19	80.73
Dry recovery (%)	22.41	11.23	10.75	14.63	14.95	95.73

GCV, Genotypic coefficient of variation; PCV, Phenotypic coefficient of variation; H^2^, Board Scene Heritability.

In the present study, broad-sense heritability (H²) varied widely among traits, ranging from 36.19% for leaf width to 97.27% for primary rhizome length. Following the scale of Robinson (1966), several traits displayed high heritability (H² > 0.60), including plant height, leaf number, leaf length, leaf area, petiole length, collar girth, number of mother rhizomes, number of primary rhizomes and the length, girth and weight of primary rhizomes as well as total yield per plant and dry recovery. These highly heritable traits suggest a greater proportion of additive genetic variance, indicating that direct selection would be highly effective for their genetic improvement. Moderate heritability (40–60%) was recorded for traits such as number of tillers per plant (53.64%) and weight of the mother rhizomes (57.88%), while leaf width showed low heritability (36.19%), signifying a prominent influence of environmental variance on this trait. Internodal length exhibited no genotypic variance, reflecting either insufficient variability among the progenies or strong environmental masking of genetic effects.

The GCV and PCV values showed a consistent pattern across traits. Traits such as number of primary rhizomes (GCV 38.82%; PCV 40.03%), weight of primary rhizomes (GCV 32.44%; PCV 40.46%), total yield per plant (GCV 29.82%; PCV 33.19%), number of mother rhizomes (GCV 24.63%; PCV 26.00%) and mother rhizome weight (GCV 22.29%; PCV 29.30%) exhibited high GCV and PCV, indicating substantial genetic variability and strong response to selection. Traits with narrow differences between GCV and PCV—such as plant height (GCV 11.00%; PCV 11.66%), leaf length (10.52% vs. 11.66%), number of leaves (10.45% vs 12.44%), leaf area (18.65% vs. 19.86%), collar girth (14.00% vs. 16.98%), length of the primary rhizome (13.64% vs13.83%), petiole length (17.51% vs. 20.99%) and dry recovery (14.63% vs 14.95%)—indicate minimal environmental influence. These traits can be improved effectively through phenotypic selection. Conversely, traits such as leaf width, where PCV substantially exceeded GCV (11.73% vs. 7.05%), reflect the greater role of environmental variation, implying slower genetic progress through direct selection.

Rhizome productivity traits displayed favorable genetic parameters. The number of primary rhizomes (H² = 94.02%), primary rhizome length (H² = 97.27%), number of mother rhizomes (H² = 89.72%), and primary rhizome girth (H² = 76.14%) exhibited highly heritability along with substantial GCV, underscoring their importance as selection criteria for yield enhancement. Similarly, total yield per plant (H² = 80.73%), and dry recovery percentage (H² = 95.73%) showed strong genetic control, suggesting their usefulness as reliable indicators of overall economic performance.

Overall, the combination of high heritability, moderate-to-high GCV and relatively narrow PCV–GCV gaps for most vegetative and yield traits demonstrates that the turmeric half-sib progeny population harbors considerable genetic potential, especially for traits linked to rhizome production. These findings strongly support the feasibility of early-generation selection for improving yield components, plant vigor, and dry recovery in turmeric breeding programs.

### 3.3 Association among traits and their contribution toward total rhizome yield per plant

**Figure 3:**
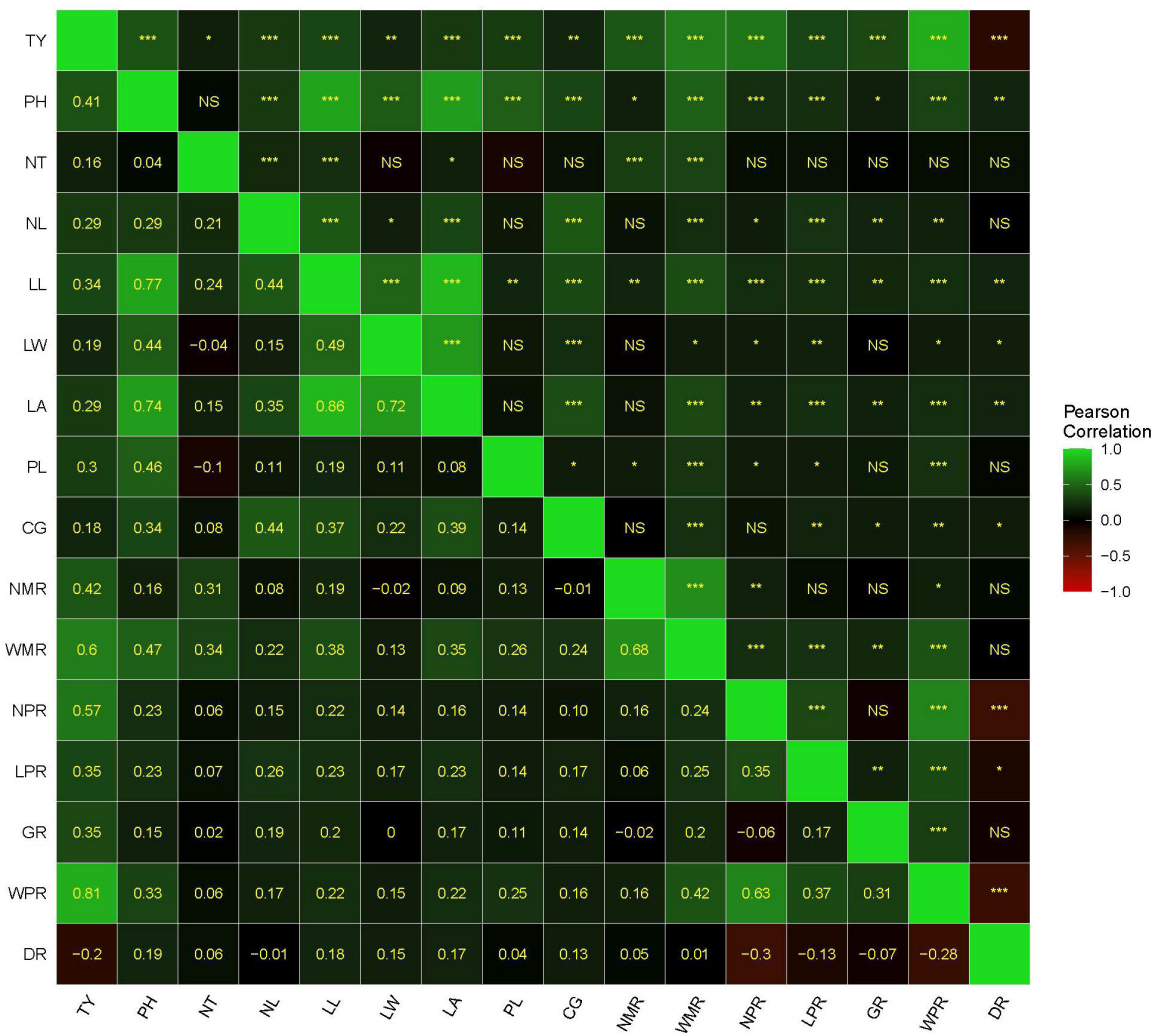
Heat map of correlation matrix for agro morphological traits among seedling progenies of turmeric. Scale on the side of the figure indicates magnitude and direction of phenotypic correlations. Shades of green from darker to lighter indicates strength of positive correlation between pairs of traits. Shades of red from darker to lighter indicates strength of negative correlation between pairs of traits. Darker to black color indicates very weak or no correlation between pair of traits. Pearson’s correlation coefficients (lower diagonal) and significance (upper diagonal) among different traits measured in this study for standardized block adjusted mean values. Significance codes: ***p ≤ 0.001, **p ≤ 0.01, *p ≤ 0.05, NS, not significant.

Pearson’s correlation coefficients among the 17 quantitative traits are presented in **Figure 3**. The observed associations reveal several biologically meaningful trait relationships that are important for selection in turmeric improvement. Significant positive correlation indicates that improvement in one trait may simultaneously enhance another, whereas significant negative correlations suggest potential trade-offs during selection.

The prime economic trait, total yield (TY), exhibited strong positive correlations with key rhizome productivity components. Notably, TY showed the highest correlation with weight of primary rhizome (WPR; r = 0.81, p ≤ 0.01), followed by weight of the mother rhizome (WMR; r = 0.60, p ≤ 0.01) and number of primary rhizomes (NPR; r = 0.57, p ≤ 0.01), highlighting these traits as the most influential contributors to yield expression in the half-sib progenies. Total yield also displayed a moderate positive association with plant height (r = 0.41) and leaf length (r = 0.34), suggesting an indirect contribution of vegetative vigor to rhizome production.

In contrast, dry recovery (DR) exhibited significant negative correlations with several rhizome and vegetative traits. Significant negative correlations were observed between dry recovery and number of primary rhizomes (r = −0.30), weight of the primary rhizome (r = −0.28), total yield (r = -0.2) and length of primary rhizomes height (r = −0.13), while weaker negative associations were recorded with girth of the primary rhizome. These trends indicate that genotypes producing larger or more succulent primary rhizomes tend to have reduced dry matter proportion, denoting a physiological trade-off between bulk yield and dry recovery, a commonly observed phenomenon in turmeric.

Vegetative traits exhibited strong internal coherence, reflecting coordinated canopy development. Plant height showed a strong positive correlation with leaf length (r = 0.77) and leaf area (r = 0.74), indicating that taller plants tend to develop larger photosynthetic surfaces. Leaf area was very strongly correlated with leaf length (r = 0.86) and leaf width (r = 0.72), confirming that laminar expansion in turmeric is jointly regulated by both longitudinal and transverse leaf growth. Moderate positive associations were also observed between petiole length and leaf area, as well as between collar girth and leaf area, suggesting that structural support traits contribute to enhanced canopy development.

Rhizome structural traits were also positively interrelated. The number of mother rhizomes (NMR) showed a strong correlation with their weight (WMR; r = 0.68), suggesting that larger mother rhizomes tend to support enhanced primary rhizome development. A similarly strong relationship was observed between the number of primary rhizomes (NPR) and the weight of primary rhizomes (WPR; r = 0.63), reinforcing the importance of both the count and size of primary rhizomes in determining overall productivity.

Moderate positive correlations were observed among several vegetative traits, including collar girth and leaf area (r = 0.39), and leaf length and leaf width (r = 0.49), indicating the collective contribution of canopy attributes to rhizome growth.

Overall, the correlation structure suggests that selection for high rhizome yield should prioritize WPR, WMR, and NPR. Simultaneously, improvement in vegetative vigor traits such as leaf length (LL), leaf width (LW), leaf area (LA) and plant height (PH) may enhance sink strength. However, due to the negative relationship of dry recovery with several yield components, a balanced selection index that accounts for both fresh yield and dry matter percentage is essential in turmeric breeding.

### 3.4 Identification of key yield-determining traits through sequential multiple regression

To identify the most influential traits governing total yield (kg plant^-1^) in turmeric seedling progenies, a sequential multiple regression analysis was performed using standardized, block-adjusted mean data. Total yield (kg plant^-1^) was treated as the dependent variable, while agro-morphological and rhizome component traits were evaluated as candidate predictors. Predictor variables were iteratively included based on minimization of Akaike Information Criterion (AIC), improvement in adjusted R², and concurrent reduction in residual standard error (RSE), ensuring model parsimony and avoid overfitting.

Sequential inclusion of predictor traits resulted in a consistent improvement in model performance (**Table 4**). The final model retained eight predictor variables, namely weight of primary rhizome (WPR), number of mother rhizomes (NMR), number of leaves (NL), girth of primary rhizome (GR), weight of mother rhizomes (WMR), number of primary rhizomes (NPR), leaf width (LW), and petiole length (PL). The progressive reduction in AIC from −280.12 to −406.23, accompanied by a steady decline in RSE (from 0.587 to 0.457), confirmed incremental gains in explanatory power with each biologically meaningful addition of traits.

**Table 4:** Sequential inclusion of predictor traits and corresponding changes in model fit statistics for total yield in turmeric seedling progenies.

Stepwise variable entry	Method	AIC	RSS	Δ Sum Sq	Residual SE	R²	Adjusted R²	P-value (trait added)
WPR	+	-280.122	90.701	173.299	0.5873	0.6564	0.6551	< 2e-16**
NMR	+	-356.30	67.529	23.173	0.5077	0.7442	0.7423	1.40E-07**
NL	+	-376.49	62.106	5.423	0.4878	0.7648	0.762	0.00131**
GR	+	-387.91	59.037	3.069	0.4765	0.7764	0.7729	2.07E-05**
WMR	+	-394.74	57.104	1.933	0.4696	0.7837	0.7795	0.00724**
NPR	+	-403.02	54.931	2.173	0.457	0.7975	0.7912	0.00192**
LW	+	-406.23	53.457	0.511	0.4582	0.7956	0.790	0.04035*
PL	+	-406.23	53.457	NA	0.457	0.7975	0.7912	0.11892^ns^

Where, WPR, Weight of primary rhizome clump^-1^ (kg); NMR, Number of mother rhizomes plant^-1^; NL, Number of leaves shoot^-1^; GR, Girth of primary rhizome (cm); WMR, Weight of mother rhizomes clump^-1^ (kg); NPR, Number of primary rhizomes clump^-1^; LW, Leaf width (cm); PL, Petiole length (cm).

The final regression model explained 79.1% of the total phenotypic variation in total yield per plant (adjusted R² = 0.791) and exhibited a low residual standard error (RSE = 0.457), indicating high predictive efficiency. The overall model was highly significant (F = 126, P< 2.2 × 10^-16^) supported by AV-Plots (**Supplementary Figure 1**). The standardized regression equation describing the relationship between total yield per plant and predictor traits is presented below:


$$TY\;\left(kg\;plant^{-1}\right)\;=-3.6e^{-11}+\;0.57\;\times \;WPR\;+\;0.22\times NMR\;+\;0.14\times GR\;+\;0.12\times NPR\;+\;0.12\;\times \;WMR\;+\;0.10\times NL\;+\;0.06\times LW\;+\;0.05\times PL$$

Among all predictors, weight of primary rhizome (WPR) emerged as the single most influential determinant of yield, accounting for 65.6% of the variation in total yield per plant (TY) when entered alone (β = 0.569, P< 0.001; **Table 5**). Subsequent inclusion of rhizome-related traits—namely number of mother rhizomes (NMR; β = 0.218), girth of primary rhizome (GR; β = 0.140), number of primary rhizomes (NPR; β = 0.122), and weight of mother rhizomes (WMR; β = 0.121)—substantially improved model fit, collectively emphasizing the dominant role of rhizome sink strength in determining yield performance.

**Table 5:** Stepwise multiple regression analysis for total yield (TY) in turmeric seedling progenies.

Predictor trait	Standardized coefficient (β)	Std. error	t-value	P-value
Weight of primary rhizome (WPR)	0.569	0.043	13.16	<0.001
Number of mother rhizomes (NMR)	0.218	0.040	5.42	<0.001
Girth of the rhizome (GR)	0.140	0.032	4.34	<0.001
Number of primary rhizomes (NPR)	0.122	0.039	3.14	0.002
Weight of mother rhizomes (WMR)	0.121	0.045	2.71	0.007
Number of leaves (NL)	0.096	0.030	3.25	0.001
Leaf width (LW)	0.060	0.029	2.06	0.040
Petiole length (PL)	0.046	0.030	1.57	0.119 (ns)

Variables were standardized prior to analysis. Trait inclusion was based on Akaike Information Criterion (AIC). Significance at P ≤ 0.05.

In contrast, vegetative-related traits contributed moderate but statistically significant effects. Number of leaves (NL; β = 0.096, P< 0.01) and leaf width (LW; β = 0.060, P< 0.05) underscored the importance of photosynthetic surface area in supporting assimilate supply. Petiole length (PL) exhibited a positive but non-significant effect (β = 0.046, P = 0.119) and was retained in the final model owing to its contribution to overall model fit (AIC), rather than individual statistical significance (**Tables 4**, **5**).

Variance inflation factor (VIF) diagnostics confirmed the absence of multicollinearity among predictors, with all VIF values remaining below 5 (range: 1.08–2.51; **Supplementary Table 2**), indicating stable and interpretable regression coefficients. The robustness of the final regression model was further evaluated through 10-fold and repeated 10-fold cross-validation. Across repeated partitions, the model maintained high predictive accuracy, with a mean RMSE of 0.481 ± 0.144, MAE of 0.360 ± 0.063, and a cross-validated R² of 0.779 ± 0.139 (**Supplementary Table 3**). These results indicate that approximately 78% of the variation in standardized total yield was consistently explained across independent data subsets, supporting the reliability of the selected predictor set for yield prediction and trait prioritization.

### 3.5 Structural equation modelling of yield architecture

**Figure 4:**
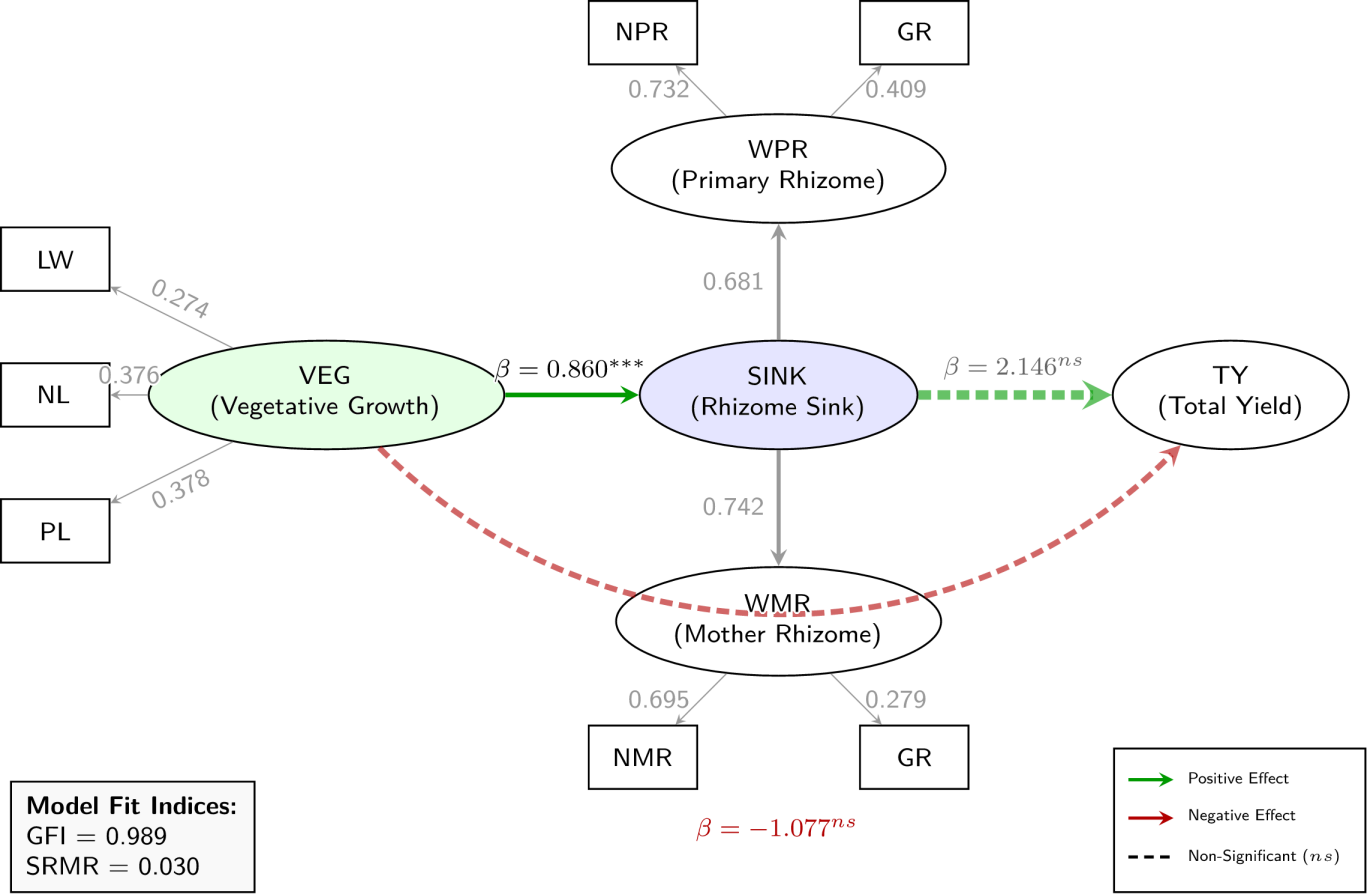
Structural equation model depicting relationships among vegetative growth, rhizome sink, and total yield (TY) in turmeric seedling progenies. Solid arrows indicate significant paths (p< 0.05), and dashed arrows indicate non-significant paths.

Structural equation modelling revealed key directional relationships underlying the yield architecture of turmeric seedling progenies (**Figure 4**). Predictor variables identified from prior multiple regression analyses were subjected to SEM using the ‘lavaan’ package in R. The hypothesized model incorporated two higher-order latent constructs—Vegetative Growth and Rhizome Sink—and two first-order latent constructs nested within Rhizome Sink. Vegetative Growth was measured by leaf width (LW), number of leaves (NL), and petiole length (PL), whereas Rhizome Sink was reflected by primary rhizome weight (WPR) and mother rhizome weight (WMR). In turn, WPR was indicated by number of primary rhizomes (NPR) and rhizome girth (GR), while WMR was indicated by number of mother rhizomes (NMR) and GR. Structurally, Vegetative Growth exerted a directly influence on Rhizome Sink, and both constructs were specified to predict total yield per plant (TY). Model estimation employed maximum likelihood with mean structures, and full information maximum likelihood was applied to handle missing data.

The model demonstrated excellent fit to the data, as evidenced by a non-significant chi-square test (*χ2 = 10.8, df = 9, p=0.290*), indicating negligible discrepancy between observed and implied covariance matrices. Supporting indices included a high goodness-of-fit index (*GFI = 0.989*) and low standardized root mean square residual (*SRMR = 0.030*), both exceeding conventional thresholds for acceptable fit and collectively confirming the adequacy of the model.

Evaluation of the measurement model confirmed reliable specification of latent constructs (**Table 6**). For Vegetative Growth, standardized factor loadings were moderate and significant for NL (Std.all = 0.376, z =3.00, p = 0.003) and PL (Std.all = 0.378, z =2.96, p=0.003), with LW serving as the reference indicator (Std.all = 0.274, z=2.41, p=0.016); These results highlights NL and PL as key contributors to canopy development and photosynthetic capacity. In case of Rhizome Sink loadings were strong and highly significant for WPR (Std.all = 0.681, z =5.12, p<0.001) and WMR (Std.all = 0.742, z =5.97, p<0.001). Lower-order indicators also showed robust contributions, including NPR on WPR (Std.all = 0.732, z =4.88, p<0.001), GR on WPR (Std.all = 0.409, z =2.91, p =0.004), NMR on WMR (Std.all = 0.695, z =4.63, p<0.001), and GR on WMR (Std.all = 0.279, z =2.02, p =0.043).

**Table 6:** Standardized factor loadings of observed traits on latent constructs in the structural equation model for yield-related traits in seedling progenies of turmeric.

Latent construct	Observed indicator	Std.all	z-value	p-value	Biological interpretation
Vegetative Growth (VEG)	Leaf width (LW)	0.274	2.41	0.016	Leaf width contributes modestly to vegetative growth, reflecting photosynthetic surface expansion
	Number of leaves (NL)	0.376	3.00	0.003	A higher number of leaves strongly represents vegetative vigor, indicating enhanced photosynthetic capacity
	Petiole length (PL)	0.378	2.96	0.003	Longer petioles significantly facilitating improved light interception and spatial leaf arrangement.
Rhizome Sink (SINK)	Primary rhizome weight (WPR)	0.681	5.12	<0.001	Primary rhizome biomass strongly reflects sink strength, and assimilation of storage of photosynthates.
	Mother rhizome weight (WMR)	0.742	5.97	<0.001	Dominant contributor to sink capacity and reserve accumulation.
Primary rhizome (WPR)	Number of primary rhizomes (NPR)	0.732	4.88	<0.001	Major driver of primary rhizome biomass through rhizome proliferation.
	Girth of rhizome (GR)	0.409	2.91	0.004	Moderate contributor to primary rhizome biomass via structural thickening.
Mother rhizome (WMR)	Number of mother rhizomes (NMR)	0.695	4.63	<0.001	Primary determinant of mother rhizome biomass.
	Girth of rhizome (GR)	0.279	2.02	0.043	Rhizome girth shows a weaker but significant contribution, mother rhizome biomass.

Standardized factor loadings (Std. all) indicate the relative contribution of observed indicators to their respective latent constructs. All reported loadings exceed the minimum threshold (0.25) considered biologically meaningful for complex quantitative traits. Values fixed for identification in the SEM estimation were subsequently standardized for interpretation.

Structural paths highlighted a dominant vegetative growth to rhizome sink linkage (standardized β=0.860, SE = 0.39, z=2.21, p=0.027; **Table 7**), signifying that enhanced vegetative vigor substantially sustains rhizome sink capacity through superior assimilate partitioning to storage organs. Direct effects on TY were non-significant: Rhizome Sink to Total yield per plant (β=2.146, SE = 1.98, z=1.08, p=0.280) trended positive, while vegetative growth to total yield per plant (β=−1.077, SE = 1.93, z=−0.56, p=0.575) was negative, implying that vegetative traits exert primary influence on yield indirectly via rhizome sink mediation. Residual variances were moderate and significant for vegetative growth indicators (LW, NL, PL), denoting substantive construct explanation, and low for rhizome sink (Std.all = 0.260), affirming predictive efficacy. Negative residuals for TY, WPR, and WMR signal potential over-parameterization or identification challenges warranting refinement. Biologically, these findings delineate a coherent yield pathway wherein early vegetative traits serve as proximal, observable proxies for subterranean rhizome sink strength, offering pragmatic selection targets in turmeric breeding.

**Table 7:** Standardized path coefficients among latent variables and total yield estimated using structural equation modelling.

Structural path	Standardized coefficient (β)	Std. error	z-value	*p*-value	Effect direction	Interpretation
Vegetative growth → Rhizome sink	0.86	0.39	2.21	0.027	Positive	Significant
Rhizome sink → Total yield (TY)	2.146	1.98	1.08	0.28	Positive	Non-significant
Vegetative growth → Total yield (TY)	−1.077	1.93	−0.56	0.575	Negative	Non-significant

### 3.6 Selection of genotypes based on selection indices

Out of 265 genotypes evaluated, all were further analyzed using MGIDI, MTSI, SH and FAI based selection indices to identify the most ideal, stable and high-performing genotypes and to compare the efficiency of these indices.

**Figure 5:**
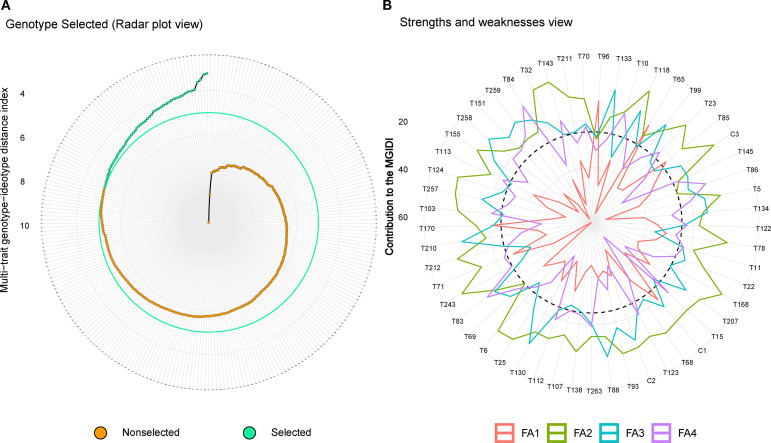
Evaluation of 265 turmeric genotypes through MGIDI in multi-environmental trials **(a)** Fifty-three genotypes were selected (represented in green dots) on the basis of 20% selection intensity using MGIDI. **(b)** Radar chart depicting the contribution of factors to MGIDI value of selected genotypes.

The MGIDI analysis retained four factors (eigenvalues >1) explaining 74.0% of total variance (**Supplementary Table 4**). FA1 (36.3%) was dominated by yield-related traits (TY, WPR, NPR), FA2 by mother rhizome traits (NMR, WMR), FA3 by rhizome girth (GR), and FA4 by vegetative traits (LW, NL, PL) (**Figure 5b**). Mean communality was high (0.74), indicating strong trait representation. At 20% selection intensity, MGIDI predicted substantial gains for TY (32.18%), WPR (45.08%), and NPR (27.45%), while constraining secondary traits (**Supplementary Table 5**). Fifty-three superior genotypes were identified, with T100, T202, T189, T196, and T231 showing the lowest MGIDI values (**Figure 5a**). Factor contribution analysis (**Figure 5b**) confirmed FA1 as the principal driver of ideotype divergence. Whereas MTSI, which integrates mean performance and stability across environments, retained four PCs explaining 75.1% variance and selected 53 stable genotypes with moderate gains for TY (15.08%) and WPR (18.42%), prioritizing adaptability over maximal gain (**Supplementary Tables 5**, **6**). SH index emphasized covariance-driven linear combinations and delivered the highest gains for individual traits (e.g., WPR 47.03%) but showed reduced biological interpretability due to negative coefficients for TY (**Supplementary Table 7**). FAI-based selection produced high gains but imposed stricter ideotype constraints on secondary traits.

Overall, MGIDI emerged as the most efficient model for capturing the underlying biological reality of turmeric yield architecture, as it maximized improvement in primary yield components while maintaining physiological balance and interpretability. MTSI was most effective for identifying broadly adapted, stable genotypes, whereas SH and FAI were more aggressive but less integrative in reflecting trait interdependence.

### 3.7 Coincidence index and common genotype selection among multi-trait stability models

**Figure 6:**
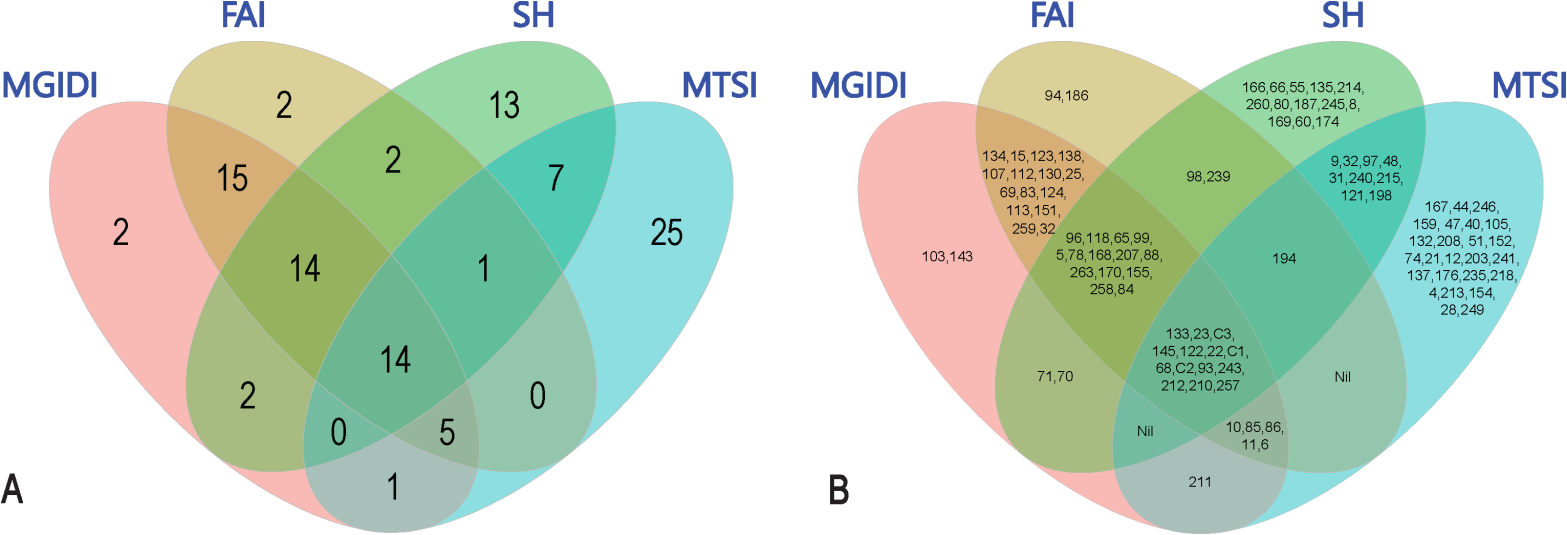
Venn diagrams showing the coincidence among various multi-trait stability models for selected genotypes **(A)** showing number of genotypes falling under **(B)** List of genotypes.

In addition to MGIDI, several multi-trait stability models like MTSI, SH and FAI were applied to identify superior genotypes at the 20% selection intensity, as illustrated in Supplementary **Figures 2**-**4**. Owing to differences in their underlying principles and computational frameworks, the sets of selected genotypes varied across indices. However, a degree of overlap among selected genotypes was observed, which is represented using a Venn diagram. The comparison among different selection indices is illustrated using a Venn diagram (**Figure 6**), depicting the coincidence index between models, where higher values indicate greater overlap of selected genotypes. Higher coincidence values indicate stronger concordance in genotype selection among methods.

Pairwise comparison revealed the highest coincidence index was observed between MGIDI and FAI, which shared 48 common genotypes (90.6%), indicating strong agreement between factor analysis–based ideotype selection and the MGIDI framework. This was followed by the overlap between Smith–Hazel and FAI, with 31 common genotypes (58.5%), suggesting substantial consistency between index-based and covariance-based selection strategies. Similarly, MGIDI and SH exhibited a relatively high coincidence (30 genotypes; 56.6%), reinforcing the agreement between these two approaches. Moderate coincidence was observed between SH and MTSI (22 genotypes; 41.5%) and between both MGIDI and MTSI as well as MTSI and FAI (20 genotypes each; 37.74%),. Importantly, a small set of 14 genotypes was consistently selected across all four indices *viz.*, C1, C2, C3, T212, T93, T257, T68, T145, T243, T23, T210, T133, T22, and T122highlighting their robust and stable multi-trait performance irrespective of the selection methodology employed. Overall, the coincidence analysis demonstrates that while all indices effectively identify superior genotypes, MGIDI and FAI show the highest level of concordance, whereas MTSI exhibits a relatively more conservative or distinct selection pattern.

## 4 Discussion

The present study systematically dissected the yield architecture and genetic potential of a substantially large population of half-sib progenies derived from five contrasting maternal accessions of turmeric. This investigation represents a pivotal advancement in turmeric breeding, addressing the long-standing limitation imposed by the species near-exclusive reliance on vegetative propagation and the resultant narrow genetic base available for sexual recombination (Anitha et al., 2025; Nair et al., 2010; Sankar et al., 2025). By integrating multi-year evaluation over two consecutive years (2024–2025) with classical quantitative genetics and advanced multivariate approaches, including structural equation modelling (SEM) and, multi-trait selection indices, the study provides robust evidence that seed-derived half-sib progenies constitute an exploitable resource for accelerating recombination breeding in turmeric.

The discussion is structured into three thematic areas: (1) genetic variability and heritability of key traits, emphasizing the generated exploitable variation; (2) dissection of yield architecture and trade-offs, focusing on trait correlations and causal relationships; and (3) efficacy of multi-trait selection indices for ideotype based genotype identification, discussing the prioritization of superior genotypes for translational breeding.

### 4.1 Genetic variability and heritability of key traits generating novel variation through sexual recombination

A fundamental premise of any successful breeding program is the availability of substantial, heritable genetic variation (σ_g_^2^) within the germplasm. The statement holds true for turmeric and has previously been demonstrated in clonal populations (Aswathi et al., 2023; Alam et al., 2024; Raghuveer et al., 2023). However, turmeric is widely regarded as recalcitrant to sexual reproduction due to ploidy-associated sterility and meiotic abnormalities, which restrict the effective recombination breeding. These hamper the judicious convergence of suitable traits under single background. The current findings provide compelling quantitative evidence that rare sexual events, leads to open-pollinated (half-sib) progenies, can successfully unlock latent genetic diversity, resulting in to significant genotypic variation (p ≤ 0.01) across 17 assessed agro-morphological, yield, and dry recovery percentage.

The pooled analysis of 265 half-sib progenies revealed wide phenotypic ranges for key economic traits. For instance, the total fresh yield per plant spanned nearly six-fold, from 0.19 kg to 1.18 kg, demonstrating profound phenotypic divergence resulting from recombination events. Similarly, dry recovery (a proxy for quality and dry matter accumulation) ranged from 14.20% to 28.80%, underscoring the potential to simultaneously improve yield bulk and processing efficiency. The markedly higher genetic variability observed in the present study can be directly attributed to the incorporation of sexual recombination through seedling-derived half-sib progenies, in contrast to earlier reports (Aarthi et al., 2022; Jayaweera et al., 2025) restricted to vegetatively propagated clonal accessions. Traditional turmeric improvement has relied almost exclusively on clonal selection from rhizome-propagated material, which inherently constrains the genetic base and limits the generation of novel allele combinations. This magnitude of variability is crucial, as it suggests that the half-sib generation—even as a product of open pollination—has successfully rearranged parental alleles into novel and potentially superior recombinant genotypes, surpassing the often-narrow limits observed in germplasm collections predominantly maintained through clonal selection (Verma et al., 2015). Trait distributions in the present seedling-derived population exhibited expansive phenotypic ranges that are highly conducive to effective selection. For instance, plant height varied from 55.0 to 128.75 cm (mean 98.15 cm), number of tillers from 1.75 to 5.50 (mean 3.12), total yield per plant from 0.19 to 1.18 kg (mean 0.50 kg), and dry recovery from 14.20 to 28.80% (mean 22.41%). Notably, 125 genotypes for plant height, 207 for number of tillers, 38 for total yield per plant, and 88 for dry recovery fell within predefined ideotypic windows proposed for turmeric improvement (Nambiar et al., 1982; Ravindran et al., 2007; Kandiannan et al., 2015). These ideotypes—moderate stature (80-110 cm PH), 4-6 tillers, TY >0.60 kg plant^-1^, DR >24%—prioritize balanced architecture for mechanical harvestability, photosynthetic efficiency, and economic recovery, mirroring elite selections like ‘Roma’ and ‘Suroma’ (5-7% curcumin, 20-25% DR) from Indian germplasm cores (Dudekula et al., 2022). The occurrence of a substantial number of genotypes approximating these ideotypic thresholds in the present seedling population underscores the effectiveness of sexual recombination in generating novel, agronomically favorable phenotypes, in contrast to the narrower phenotypic spectrum typically observed in clonal turmeric collections.

The utility of present observed variability hinges on its heritable nature. High broad-sense heritability (H^2^ > 0.60) for most traits indicates that a large proportion of the phenotypic variance (σ_p_^2^​) is attributable to genetic factors (σ_g_^2^​), thus enabling effective genetic gain through phenotypic selection (Bernardo, 2020; Saroj et al., 2021; Kapoor et al., 2026). Notably, high heritability observed for key yield components: total yield per plant (80.73%), dry recovery % (97.27%), weight of primary rhizomes (64.3%), number of primary rhizomes (94.02%), and length of primary rhizome (97.27%). Such high board-sense heritability for these economically important traits suggested that the superior performance of selected progenies is largely due to inherent genetic makeup, offering high predictability for trait inheritance in subsequent clonal generations (Raghuveer et al., 2023).

Furthermore, traits exhibiting high genotypic coefficient of variation (GCV) coupled with high heritability are prime targets for effective genetic selection (Robinson, 1966). Key yield components—specifically, number of primary rhizomes (GCV = 38.82%), weight of primary rhizomes (GCV = 32.44%), and total yield per plant (GCV = 29.82%)—all exceeded the 20% threshold, confirming strong selection potential. Importantly, the narrow disparity between GCV and Phenotypic Coefficient of Variation (PCV) for high heritable traits (e.g., total yield: GCV = 29.82% vs. PCV = 33.19%; dry recovery: GCV = 14.63% vs. PCV = 14.95%) reinforces that environmental influence on these characters is minimal. This combination of favorable genetic parameters substantiates the use of phenotypic selection in early generations of seed-derived turmeric populations, thereby streamlining the breeding process. Consequently, previous studies typically reported only moderate genotypic coefficients of variation for yield and rhizome traits (often in the range of 10–22%), with corresponding moderate heritability (≈60–80%) and limited expected genetic advance, reflecting the narrow scope for selection response in clonally structured populations (Jayaweera et al., 2025).This contrasts with previous breeding efforts reliant solely on clonal selection or mutation breeding, where genetic progression is often incremental (Mukesh Sankar et al., 2025).

### 4.2 Dissection of yield architecture and trade-offs through understanding causal pathways

Rhizome yield in turmeric is a complex, integrative trait governed by multiple morphological and physiological processes, including vegetative growth, tillering capacity, leaf architecture, and rhizome sink development. Understanding the interrelationships among traits is crucial for developing efficient indirect selection strategies, particularly in crops like turmeric where the economic product (rhizome) is subterranean and phenotyping for yield components can be labor-intensive. The analysis of Pearson’s correlations, sequential multiple regression models and, more definitively, the Structural Equation Model (SEM), provided a clear hierarchical framework of yield contribution (Grace et al., 2016; Zhu et al., 2024). Although SEM has gained prominence in crop physiology and phenomics. its application in spice crops, and particularly in turmeric, remains extremely limited. Traditional correlation and regression analyses have been widely used to study trait associations in turmeric, but these methods often fail to capture the hierarchical and directional relationships among traits.

#### 4.2.1 Direct and indirect yield determinants

Correlation analysis established that total yield (TY) is overwhelmingly driven by subterranean *sink* strength components. The highest positive associations were observed between TY and weight of the primary rhizome (WPR; r=0.81, p ≤ 0.01), followed by weight of the mother rhizome (WMR; r=0.60), and number of primary rhizomes (NPR; r=0.57). This confirms WPR as the single most critical trait for yield improvement, an observation strongly supported by stepwise regression, which identified WPR as the primary predictor (adjusted R^2^ = 0.791) which was also reported by Aarthi et al. (2022) and Praneetha et al. (2020). The prominent role of primary rhizome mass and number in determining yield is consistent with earlier reports where positive and significant associations between yield and the number of mother rhizomes per plant have been reported by Jagadeeshkanth et al. (2017); Mamatha et al. (2020), and Singh et al. (2018). Likewise, several studies have demonstrated that the number of primary rhizomes per plant is a major contributor to total rhizome yield (Prajapati et al., 2014; Luiram et al., 2019; Poonam and Jakhar, 2022; Jayaweera et al., 2025). These findings align with general physiological principles in storage-organ crops, where the size and quantity of the primary storage unit dictate final economic output (Smith et al., 2018; Sahoo et al., 2025).The strong positive relationships among the rhizome traits (e.g., NPR and WPR, r=0.63; NMR and WMR, r=0.68) suggest that selecting for bolder primary rhizomes that are also numerous will synergistically boost overall yield.

Beyond the direct rhizome components, vegetative traits (representing the *source* capacity) were found to play a vital, indirect role. Vegetative vigor, as measured by traits like plant height (PH), leaf length (LL), and leaf area (LA), showed moderate positive associations with total yield (e.g., TY−PH, r=0.41). The SEM provided a more nuanced view of this relationship, confirming that vegetative growth (modelled via leaf width, leaf number, and petiole length) strongly influences rhizome sink strength (β=0.860, p=0.027), which, in turn, mediates the ultimate effect on total yield. In line with this, Jayaweera et al. (2025) reported the number of leaves per plant exhibited a positive direct effect on fresh rhizome yield (direct effect = 0.023) and showed a significant positive correlation with yield (r = 0.293, p ≤ 0.01). Leaf petiole length was also positively associated with total rhizome yield (Jayaweera et al., 2025). Earlier studies have similarly reported positive influences of leaf number on rhizome yield (Mamatha et al., 2020; Dev and Sharma, 2020; Jagadeeshkanth et al., 2017; Vimal et al., 2018; Poonam and Jakhar, 2022), while leaf width has been identified as a favorable contributor to yield in turmeric (Mamatha et al., 2020; Jayaweera et al., 2025). Similar positive associations of rhizome yield with morphological attributes such as plant height and leaf area have also been documented in ginger, underscoring the contribution of photosynthetic efficiency and assimilatory capacity to rhizome development (Dev and Sharma, 2020). This structural relationship validates the use of easily observable above-ground traits as early-stage proxies for predicting future subterranean yield potential in large-scale seedling screens. This is a crucial practical implication for breeders, allowing for the culling of low-performing genotypes based on vegetative metrics before the resource-intensive process of final rhizome harvest and yield measurement.

#### 4.2.2 The yield-quality trade-off

A key physiological and breeding challenge identified was the significant negative correlation between total yield and dry recovery percentage (DR). Dry recovery, which reflects the dry matter and starch content, showed significant negative correlations with bulk yield components: NPR (r=−0.30), WPR (r=−0.28), and TY (r=−0.20). This established trade-off suggests that genotypes optimized for maximizing fresh yield often do so by accumulating more water or more succulent tissues, leading to a dilution effect on dry matter percentage and, frequently, quality components (Praneetha et al., 2020; Tejavathi et al., 2020). This common scenario in root and rhizome crops necessitates a shift from single-trait selection (e.g., selecting solely for maximum yield) to an integrative, multi-trait selection framework that penalizes excess succulence while rewarding balanced performance. The observed ideotype distribution, where high-yielding genotypes did not uniformly cluster in the highest dry recovery classes, further underscores the necessity of a balanced approach to capture both bulk and quality (Duraisamy et al., 2025).

### 4.3 Efficacy of multi-trait selection indices for ideotype identification

Given the complexity of turmeric yield architecture, characterized by multiple positively correlated yield components and a trade-off with the crucial quality trait of dry recovery, the utility of single-trait selection methods is severely limited. Conventional breeding often struggles with this multivariate challenge, leading to incremental rather than transformative genetic gain. To overcome this, the present study employed four contemporary multi-trait selection indices—Multi-Trait Genotype-Ideotype Distance (MGIDI), Multi-Trait Stability Index (MTSI), Smith-Hazel (SH), and Factor Analysis-Ideotype (FAI)—to integrate information from selected and associated vegetative, rhizome, and yield traits into a unified selection framework.

The application of these indices was highly effective, collectively identifying 53 superior half-sib progenies each (representing the 20% selection intensity) across all resulting common 14 genotypes (say, C1, C2, C3, T212, T93, T257, T68, T145, T243, T23, T210, T133, T22, and T122(. These selections were not merely high-yielding but represented a balanced ideotype, prioritizing yield, sink capacity, and stability. The identification of common selections across the diverse methodologies underscores the robustness and consensus among these advanced statistical tools. Comparable findings were reported in pearl millet (Kapoor et al., 2026); black pepper (Shivakumar et al., 2022) and ginger (Begum et al., 2023), where multi-trait indices coupled with factor analysis effectively captured inter-trait relationships and improved the precision of genotype selection across yield and quality parameters.

### 4.4 Integration of yield components and management of trade-offs

A key outcome of the present study is the clarification of how vegetative traits and rhizome traits are integrated to determine total yield in turmeric, and how inherent physiological trade-offs can be effectively managed through multi-trait selection. Structural equation modelling (SEM) revealed that vegetative growth—defined as a first-order latent construct measured by leaf width (LW), number of leaves (NL), and petiole length (PL) exerts a strong and statistically significant positive influence on rhizome sink strength (β = 0.860, *P* = 0.027). This relationship indicates that seedlings with superior canopy development tend to establish a more robust underground sink, thereby facilitating assimilate allocation to storage organs.

Rhizome sink was specified as a higher-order latent construct reflected by weight of primary rhizomes (WPR) and weight of mother rhizomes (WMR). Among these, WMR showed a strong and highly significant loading (β = 0.742, P< 0.001), underscoring the dominant role of mother rhizome biomass in defining sink strength. At the lower hierarchical level, WPR was measured by number of primary rhizomes (NPR) and girth of primary rhizome (GR), while WMR was measured by number of mother rhizomes (NMR) and GR, reinforcing the interpretation of sink capacity as an integrated outcome of both rhizome number and size attributes. The low residual variance of the rhizome sink construct further indicates that vegetative growth explains a substantial proportion of sink development.

In contrast, neither vegetative growth nor rhizome sink exhibited a statistically significant direct effect on total yield (TY). The direct path from vegetative growth to TY was negative and non-significant (β = −1.077, *P* = 0.708), while the direct effect of rhizome sink on TY was positive but also non-significant (β = 2.146, *P* = 0.480). Collectively, these results demonstrate that vegetative vigor influences yield primarily through its indirect effect mediated by rhizome sink development, rather than through a strong independent direct contribution to final yield. This mediated pathway provides a biologically coherent explanation for yield determination in turmeric, where above-ground vigor acts as an upstream driver of subterranean biomass accumulation. This framework highlights the value of early-season vegetative traits as reliable, non-destructive proxy indicators for selecting superior genotypes, particularly in turmeric where the economic product develops below ground. Importantly, several vegetative traits used in the model exhibited high heritability (e.g., number of leaves, H² = 70.60%; petiole length, H² = 69.54%), further supporting their utility in accelerating selection decisions in early breeding generations.

While total yield exhibited strong positive correlations with primary rhizome weight (r=0.81), mother rhizome weight (r=0.60), and primary rhizome number (r=0.57), it maintained a detrimental negative correlation with dry recovery (r=−0.20). Selection based purely on fresh yield would inevitably compromise quality. The multi-trait indices circumvented this issue by assigning appropriate weights or penalizing genotypes that deviated significantly from the desired ideotype threshold for dry recovery (which was set to surpass 24% for optimal quality).

For instance, the Smith-Hazel (SH) index, which explicitly incorporates phenotypic and genetic covariances among traits, demonstrated remarkable effectiveness in balancing these competing selection objectives. Selection gain (SG) analysis using the SH index revealed the highest predicted genetic gains for weight of primary rhizomes (WPR) (47.03%) and total yield (TY) (27.82%), while simultaneously achieving positive selection differentials across all traits, including dry recovery. This confirms that the SH index successfully exploited the high additive genetic variance present in the progeny population, particularly for highly heritable traits such as WPR (H^2^ = 64.3%) and TY (H^2^ = 80.73%).

### 4.5 Convergence among diverse selection indices reveals robust ideotype-based selection

Among the indices, MGIDI delivered the highest predicted genetic gain for total yield (32.2%), closely followed by FAI (31.4%), whereas SH and MTSI resulted in comparatively moderate gains (22.5% and 15.1%, respectively). The success of the MGIDI and FAI indices, reflected in their high coincidence index, stems from their ability to translate complex phenotypic data into a quantifiable distance from a defined ideotype, thereby enabling breeders to select for an optimal trait profile rather than maximum performance in a single trait (Donald, 1968; Rocha et al., 2018; Olivoto et al., 2019). In addition, both approaches leveraged insights derived from the Structural Equation Model (SEM) analysis, which provided a hierarchical understanding of the yield architecture.

The comparatively lower gain achieved by the Smith–Hazel (SH) index, despite its foundational role in quantitative genetics, can be attributed to its sensitivity to multicollinearity among traits and its reliance on accurate estimates of genetic and phenotypic variance–covariance matrices, which are often unstable in early-generation breeding populations (Hazel, 1943; Cerón-Rojas and Crossa, 2018). Similarly, the moderate performance of the MTSI reflects the inherent trade-off between stability-focused selection and yield maximization, as stability-oriented indices tend to penalize high-performing but variable genotypes (Rocha et al., 2018).

## 5 Conclusion

The results decisively validate the application of multi-trait selection indices in turmeric breeding, particularly when utilizing genetically diverse seedling progenies. These indices provide a robust, comprehensive, and objective framework for ideotype identification, overcoming the limitations of conventional sequential selection approaches. By effectively balancing high yield potential with stability, these methods establish a strong foundation for rapid and efficient recombination-based improvement in turmeric.

## Data Availability

The original contributions presented in the study are included in the article/**Supplementary Material**. Further inquiries can be directed to the corresponding authors.
