# Supplementary figures and images for: Dissecting the yield architecture and selecting superior seedling-derived half-sib progenies of turmeric (Curcuma longa L.) through multi-trait selection indices
Source: Front Plant Sci. 2026 May 5;17:1812796. doi: 10.3389/fpls.2026.1812796 (PMC13183827; doi:10.3389/fpls.2026.1812796)

# Added-Variable Plots

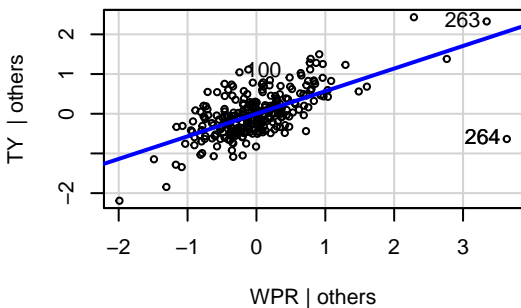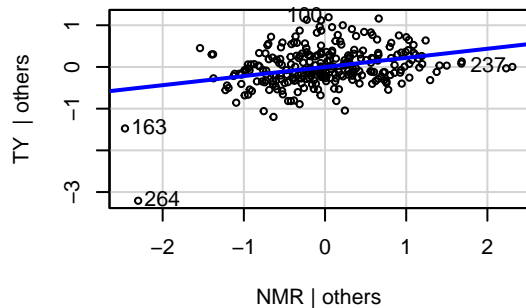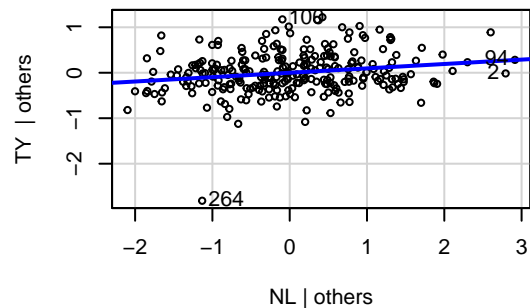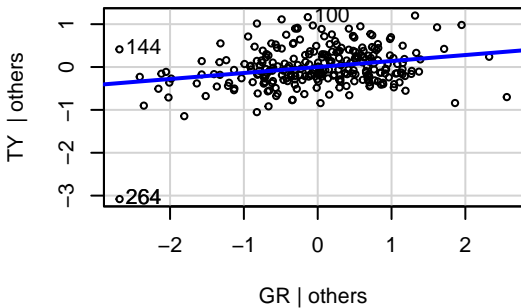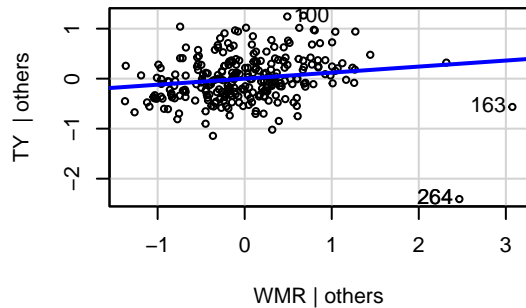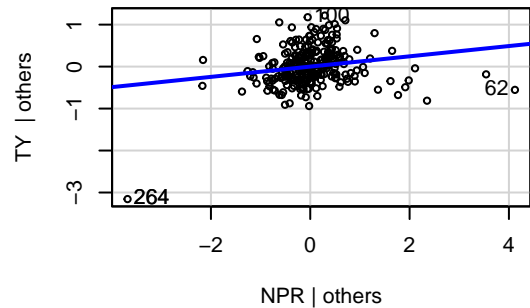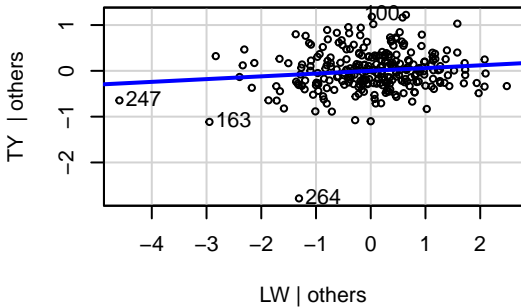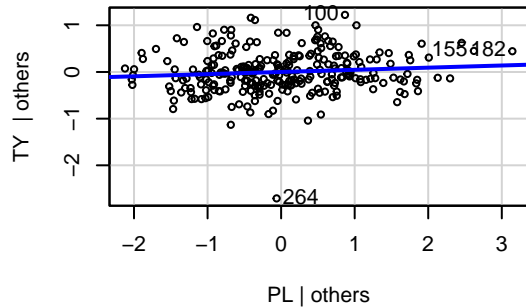

Supplement: Supplementary file 1 [file DataSheet1.pdf]

# Radar Plot (Smith-Hazel Index)

Individual genetic worth

70  
60  
50  
40

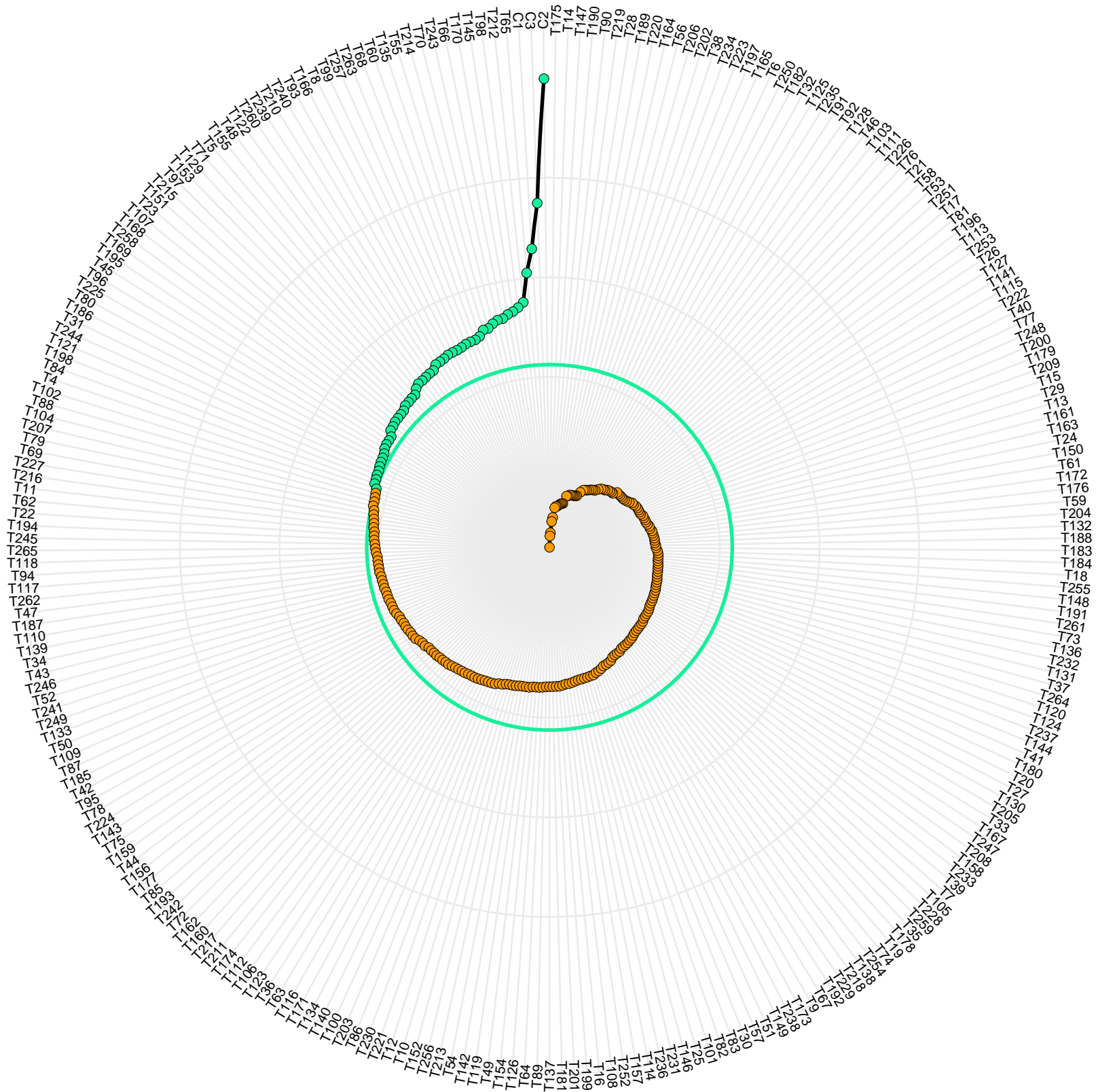

● Nonselected      ● Selected

Supplement: Supplementary file 2 [file DataSheet2.pdf]

# Multitrait stability index (MTSI)

Radar Plot of selection

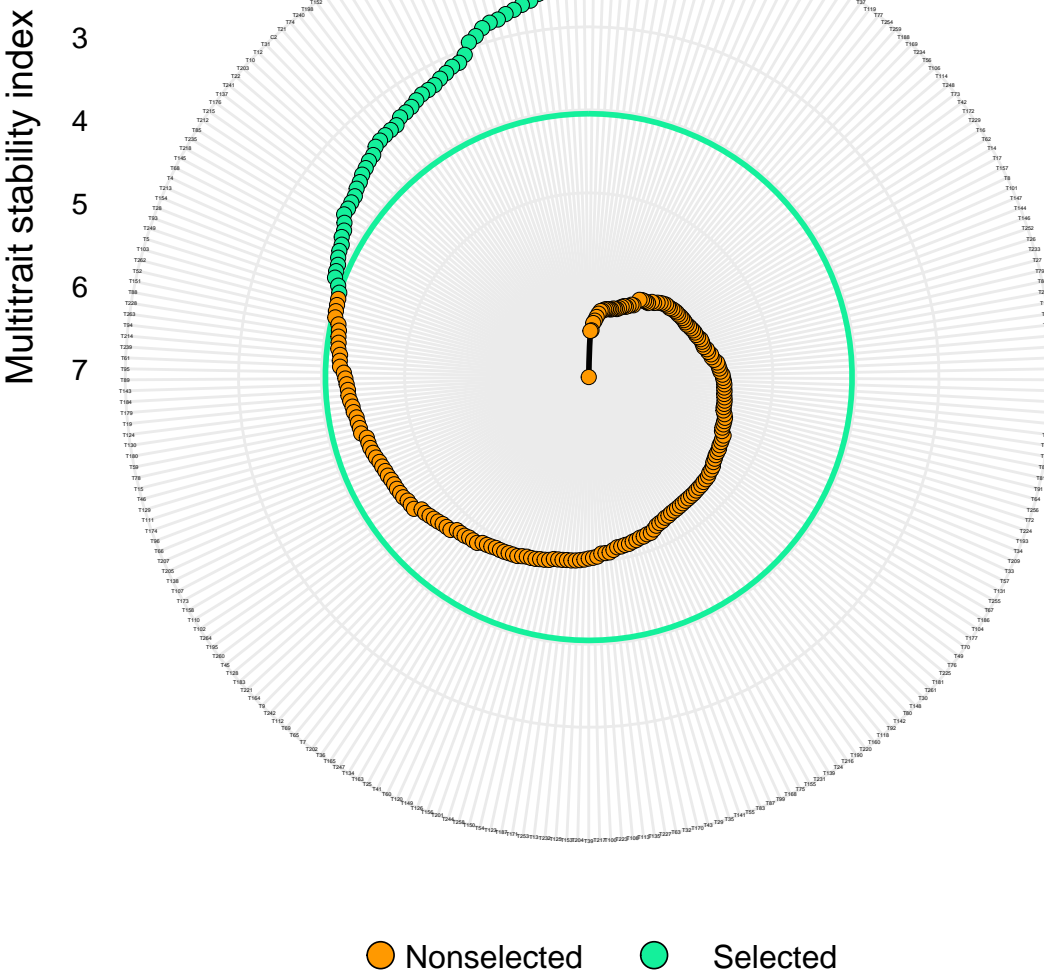

Strengths and weaknesses view

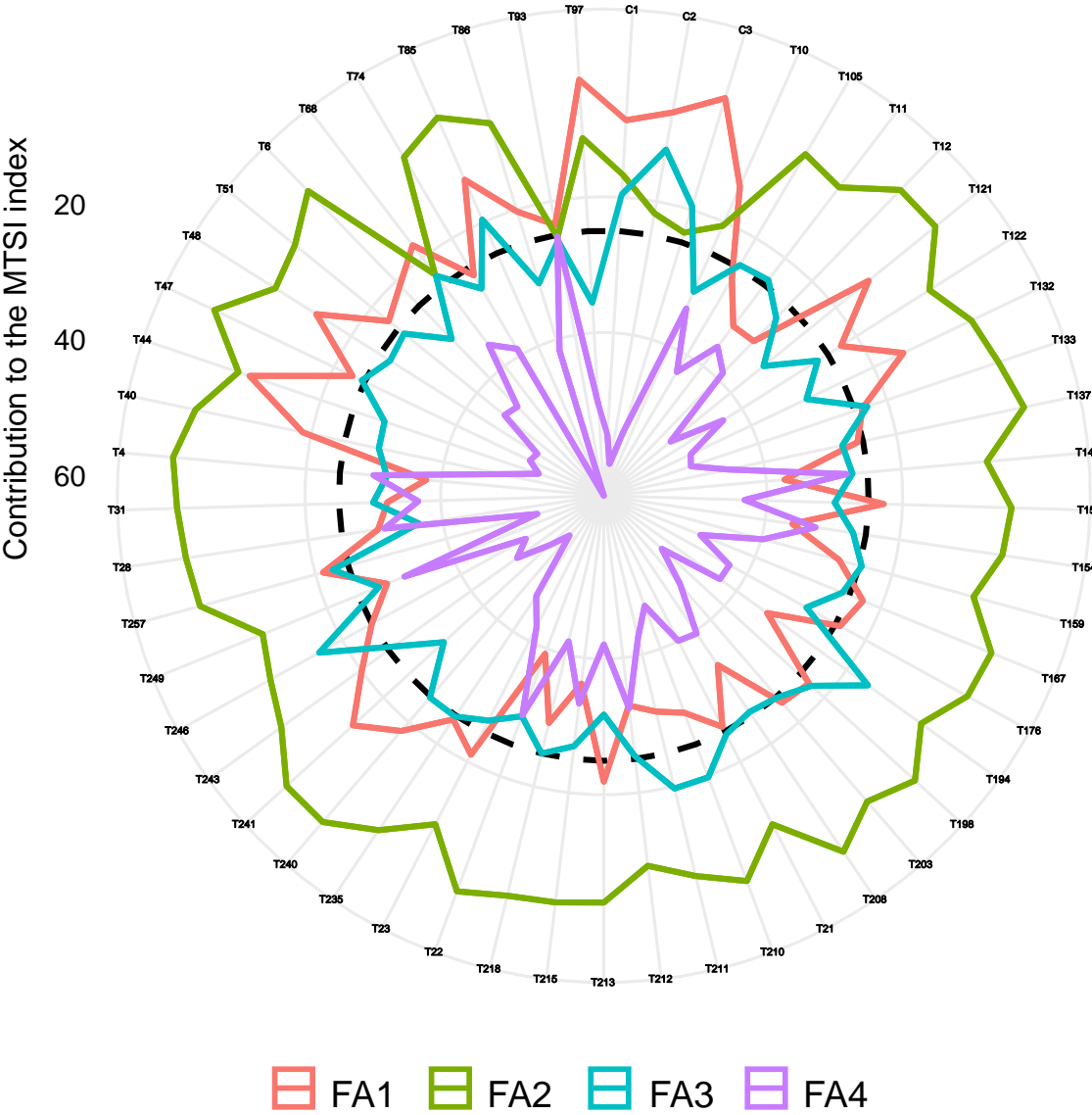

Supplement: Supplementary file 3 [file DataSheet3.pdf]

Factor Analysis-Ideotype (FAI)

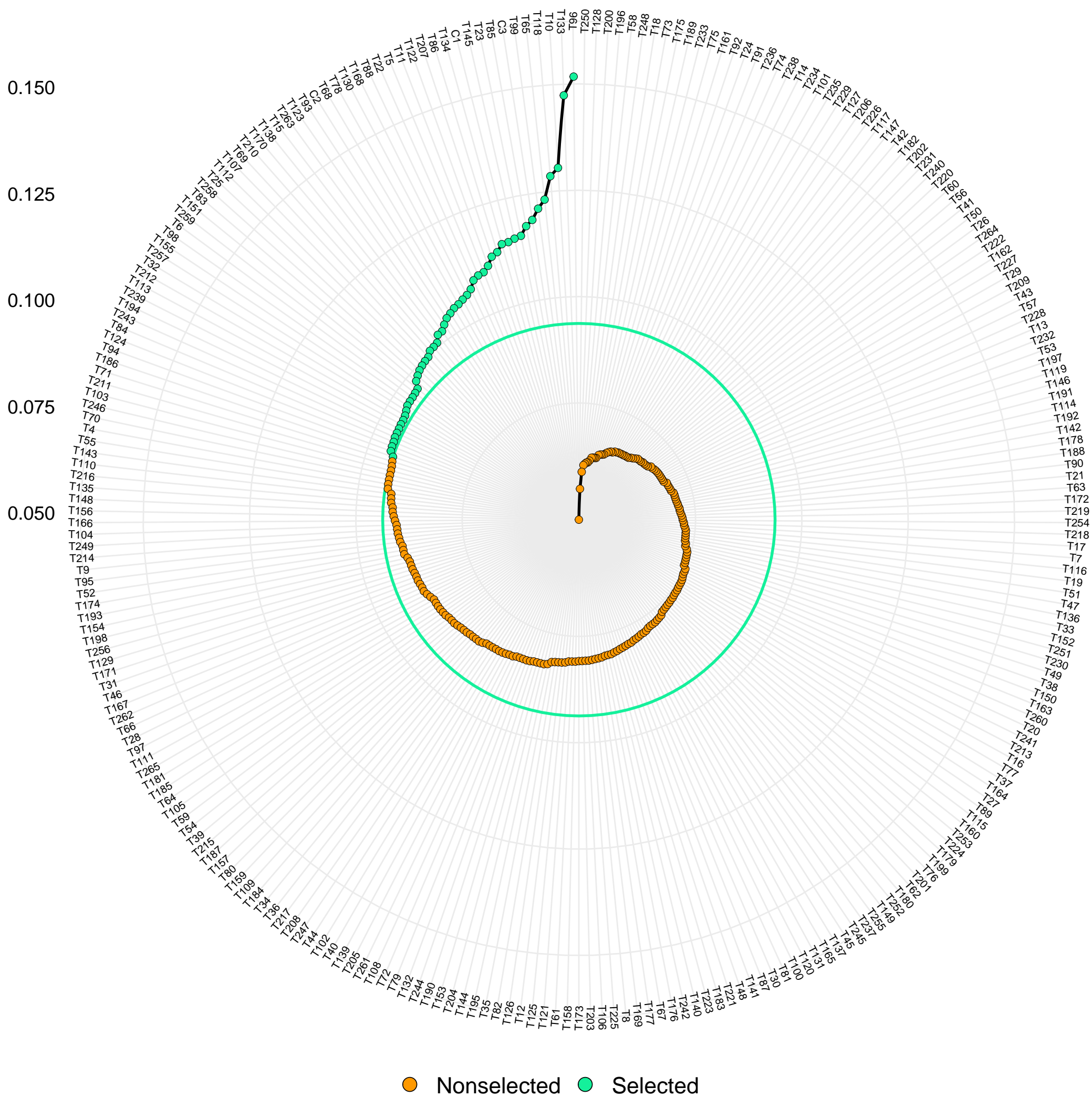

Supplement: Supplementary file 4 [file DataSheet4.pdf]
